# The piezochiral effect

**DOI:** 10.1038/s41586-026-10845-5

**Published:** 2026-07-29

**Authors:** Z. Zeng, M. Först, M. Fechner, X. Deng, A. Cavalleri, P. G. Radaelli

**Affiliations:** 1 Max Planck Institute for the Structure and Dynamics of Matter, Hamburg, Germany; 2 Department of Physics, Clarendon Laboratory, University of Oxford, Oxford, UK

**Keywords:** Structure of solids and liquids, Structure of solids and liquids

## Abstract

Chirality is a pervasive property of matter that underpins many important phenomena across physics^1^, chemistry^2^ and biology^3^. Given its broad importance, the development of protocols for rational control of chirality in solid-state systems is highly desirable, especially if this effect can be tuned continuously and in two directions. Yet, this goal has remained elusive owing to the absence of a universal conjugate field that couples linearly to this structural order^4–6^. Here we introduce the piezochiral effect, which enables control of chirality through mechanical strain. We show by symmetry analysis that uniaxial strain induces chirality in a broad class of achiral crystals that host fragments of opposite chirality within each unit cell^7,8^, an effect that has so far remained unrecognized. The strain-induced handedness can be tuned either by changing the strain direction or by switching between compressive and tensile strain. We experimentally verify this effect in AgGaS_2_, using measurements of the optical activity under strain. Our discovery establishes a new scheme for chirality control, with potential applications that range from spintronics to asymmetric catalysis, and enantioselective interactions in biosystems.

## Abstract (web summary)

The piezochiral effect, a new member of the family of strain-responsive functionalities alongside piezoelectricity and piezomagnetism, is introduced, enabling control of chirality through mechanical strain, with future applications in photonics, spintronics, biosensing and quantum information.

## Main

Chirality, the geometric property of an object that cannot be superimposed onto its mirror image^9^, plays a central role across the physical and life sciences. It forms the basis of a wide range of functionalities, including magneto-chiral anisotropy^1^, asymmetric synthesis^2^ and enantioselective drug action^3^. Given its importance, extensive efforts have been devoted to the design and realization of chiral materials, primarily through bottom-up approaches, including crystal growth^10^, molecular synthesis^2^ and nanostructure fabrication^11^.

An alternative strategy is to explore the control of chirality by external stimuli. Material systems near an achiral–chiral phase transition have received particular attention^12–15^, as their inherent instability makes them susceptible to perturbations such as strain or pressure^5,16–18^. However, existing approaches merely modulate pre-existing chirality and do not enable the induction of chirality with a deterministic preset of the resulting handedness.

Only recently it has been shown that chirality with selected handedness can be induced in an achiral system through the excitation of specific phonon modes using terahertz-frequency optical pulses^19,20^. In this case, the sign of the induced chirality was set by the polarization of the light field. Axially ordered solids are another promising class of material, as the axial state enables the direct coupling between chirality and electric fields^21,22^. Still, the realization of a general coupling between mechanical strain and chirality, referred to as piezochirality, is highly motivated and has been anticipated in the past^23^. Here we establish a rigorous, general formalism of the piezochiral effect, which provides a new approach for the deterministic control of chirality applicable across a broad class of materials.

## Material classes with a piezochiral effect

Strain engineering has long been recognized as a powerful means to tailor material functionalities^24–27^. Well-established examples, such as piezoelectricity^28^ and piezomagnetism^29^, have been extensively studied and led to widespread applications^30^. However, the linear coupling of strain to chirality has not yet been investigated. Whereas strain, polarization and magnetization are directional quantities, chirality is a non-directional pseudoscalar that reverses sign under inversion symmetry^31^. This distinction makes the coupling between strain and chirality elusive.

**Fig. 1:**
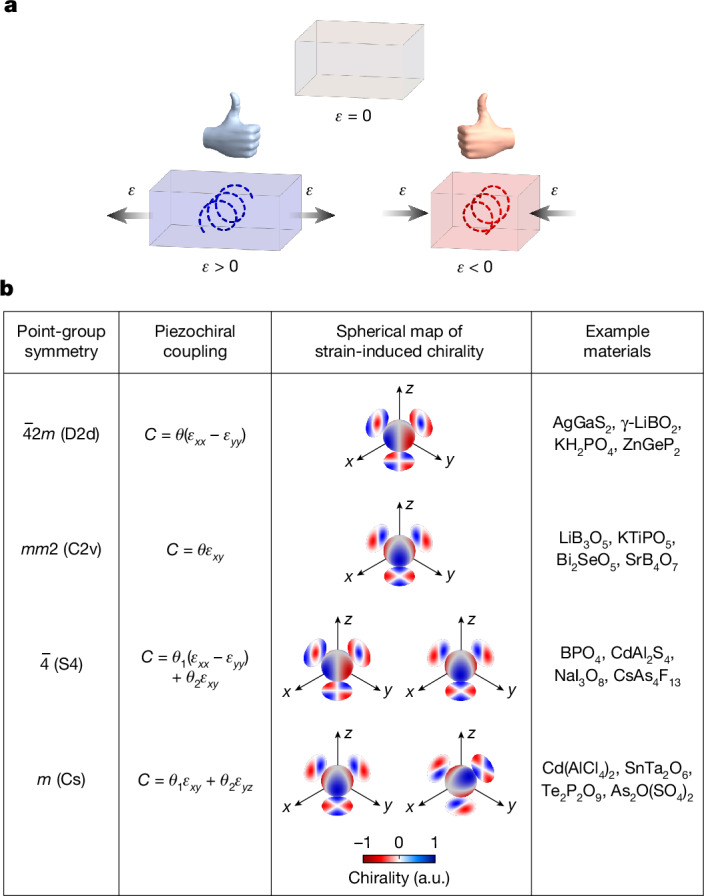
The piezochiral effect. **a**, An achiral system at equilibrium develops chirality under strain. Switching between tensile strain and compressive strain reverses the handedness of the induced chirality. **b**, Piezochiral coupling forms. From left to right, four achiral point groups supporting coupling between strain and chirality, their respective coupling forms, visualization of the coupling forms and representative materials. a.u., arbitrary units.

The piezochiral effect is defined here as the induction of chirality in a system by the application of strain (Fig. 1a). Mathematically, the effect can be written as

1 $$C=\sum _{{ij}}{T}_{{ij}}{\varepsilon }_{{ij}}$$

in which *C* denotes the strain-induced chirality, *ε*_*ij*_ is the strain tensor and *T*_*ij*_ is the piezochiral tensor, which we introduce here to describe the coupling between strain and chirality (Supplementary Information). Notably, in contrast to many conventional rank-2 tensors, such as the strain tensor, which are even under spatial inversion, the piezochiral tensor changes sign under this symmetry operation.

In this study, we focus on the piezochiral effect in achiral systems, in which reversing the sign of strain by switching between compressive and tensile strain allows controlled generation of both left- and right-handedness, analogous to the reversed electric polarization and magnetization in piezoelectricity and piezomagnetism, respectively. Building on this formalism, we performed a systematic group theory analysis. We enumerated all of the achiral crystallographic point groups and identified the cases in which the piezochiral effect is symmetry-allowed (Fig. 1b). For each allowed group, we derived the explicit form of the piezochiral coupling consistent with the symmetry constraints (Supplementary Tables 1–4) and found that it breaks all of the rotoinversion operations to drive the transition from an achiral to a chiral state (Supplementary Table 5).

As a clear example, we consider the point group $\bar{4}2m$ (D2d), which is achiral in its unstrained equilibrium state. When external strain is applied, the symmetry of the system is lowered to a subgroup in which chirality becomes allowed. In this case, the induced chirality can be described by the coupling

2 $$C=\theta ({\varepsilon }_{{xx}}-{\varepsilon }_{{yy}})$$

in which *θ* is a material-specific coupling coefficient and *ε*_*xx*_ and *ε*_*yy*_ represent externally applied strain components.

This coupling relation suggests that chirality can be induced in this point group by applying uniaxial strain along either the *x* or the *y* direction. Because *ε*_*xx*_ and *ε*_*yy*_ enter the coupling with opposite signs, applying the same type of strain (compressive or tensile) along the two directions induces chirality of opposite handedness.

By symmetry, the piezochiral coupling is also allowed in chiral systems, in which it can modulate existing chirality (Supplementary Tables 6–10). Also, the framework of the piezochiral effect generalizes to higher orders, at which chirality couples to quadratic or even higher order strain terms. This behaviour is directly analogous to higher-order piezoelectricity^32^ and higher-order piezomagnetism^33^ and becomes especially important in materials in which the linear term is symmetry-forbidden.

The general form of the higher-order couplings can be expressed as

3 $$C=\sum _{{ij}}{T}_{{ij}}{\varepsilon }_{{ij}}+\sum _{{ijkl}}{Q}_{{ijkl}}{\varepsilon }_{{ij}}{\varepsilon }_{{kl}}+\cdots $$

**Extended Data Fig. 1:**
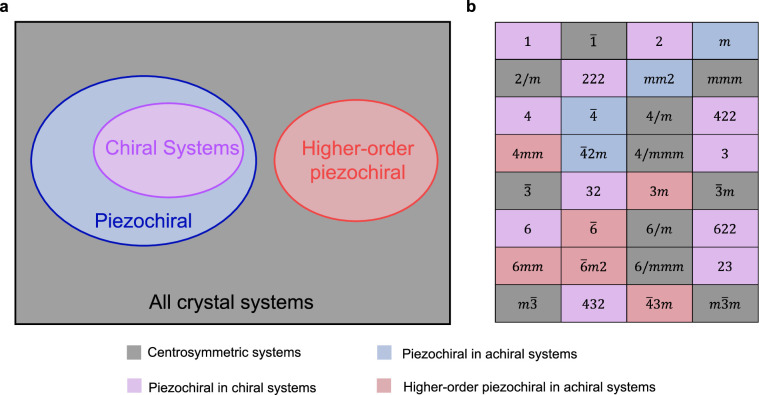
Classification of crystal systems. **a**, Piezochiral systems form a subset of non-centrosymmetric systems, including all chiral systems and certain achiral systems. **b**, Among the 32 crystallographic point groups, 11 are centrosymmetric (grey), 11 are chiral and inherently piezochiral (purple), four are achiral yet piezochiral (blue) and six are achiral and support only higher-order piezochirality (red).

Through an analysis of all achiral crystallographic point groups, we identify four groups in which second-order piezochiral coupling provides the leading contribution (Supplementary Table 11), including materials such as GaSe (point group $\bar{6}m2$). Also, two groups allow third-order piezochiral coupling as the leading term (Supplementary Table 12), including materials such as GaAs (point group $\bar{4}3m$). A summary of the classification is shown in Extended Data Fig. 1.

## Experimental verification in the model system AgGaS_2_

**Fig. 2:**
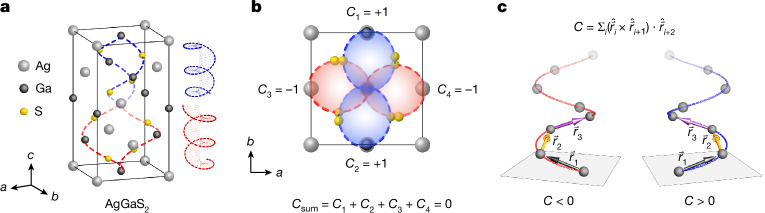
Static chiral order parameter in AgGaS_2_. **a**, Unit cell of the antiferrochiral crystal AgGaS_2_, composed of chiral substructures with opposite handedness. **b**, Normalized chiral order parameter of AgGaS_2_. Substructures of opposite handedness yield opposite values, resulting in a vanishing net chiral order parameter. **c**, Definition of the chiral order parameter as the sum of triple products formed from normalized vectors connecting neighbouring atoms along the spiral.

We investigate the model system silver gallium sulfide (AgGaS_2_, point group $\bar{4}2m$) to uncover the microscopic origin of the piezochiral effect at the unit-cell level. The crystal structure of AgGaS_2_ contains chiral substructures of opposite handedness within each unit cell (Supplementary Table 13), shown in red and blue in Fig. 2a. These substructures have an antiferrochiral arrangement, in which the opposite handedness compensates, resulting in an overall achiral structure^7,8^.

Next we define a geometrical order parameter to quantitatively describe the chirality in the crystal. We choose a pseudoscalar defined as the sum of the triple products formed by the normalized vectors connecting four neighbouring atoms in a spiral structure^34^ (Fig. 2c).

4 $$C=\sum _{i}\left({\hat{\mathop{r}\limits^{\rightharpoonup }}}_{i}\times {\hat{\mathop{r}\limits^{\rightharpoonup }}}_{i+1}\right)\cdot {\hat{\mathop{r}\limits^{\rightharpoonup }}}_{i+2}$$

This order parameter serves as an effective measure of chirality, vanishing in an achiral structure, changing sign for left- and right-handed enantiomers and remaining invariant under rotations. In AgGaS_2_, the order parameter is evaluated for all of the substructures shown in Fig. 2a,b, showing opposite values for the substructures of opposite handedness. The sum of the chiral order parameters of these substructures yields zero, indicating an overall achiral unit cell (Fig. 2b).

**Fig. 3:**
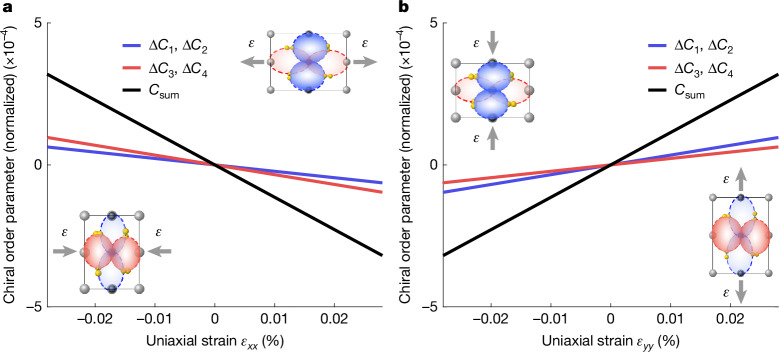
Calculation of strain-induced chirality in AgGaS_2_. **a**, Evolution of chirality in the chiral substructures and in the overall unit cell under uniaxial strain along the *x* direction. **b**, Same as **a** but under uniaxial strain along the *y* direction. Source data

Next we used density functional theory calculations to assess the emergence of chirality in AgGaS_2_ under uniaxial strain. The strain was introduced by constraining the lattice parameter along the *a* or *b* axes, followed by full relaxation of the remaining lattice parameters and all of the internal atomic coordinates. The chiral order parameter was then calculated from the relaxed structures (Supplementary Information), revealing that uniaxial strain along the *x* direction modulates the chirality amplitude in both of the opposite-handedness substructures (Fig. 3a). Notably, the degeneracy between the left- and right-handed substructures is lifted, yielding a finite net chirality of the unit cell, with its handedness determined by the sign of the applied strain. The lifted degeneracy is reflected in a view of the AgGaS_2_ crystal along its *c* axis, which clearly shows the red and blue substructures distorted into different shapes (Fig. 3a inset). When strain is applied along the orthogonal* y* direction, the sign of the induced chirality is reversed (Fig. 3b), consistent with the symmetry-derived form of the piezochiral coupling discussed above.

**Fig. 4:**
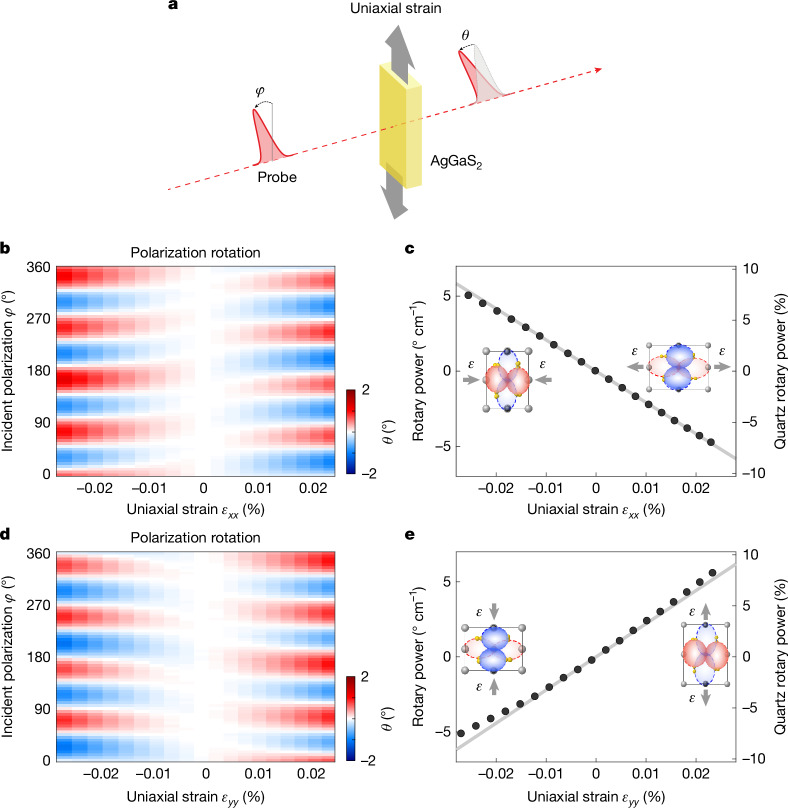
Observation of piezochiral effects. **a**, Schematic of the experiment. A linearly polarized optical pulse, propagating along the crystal *c* axis, probes strain-induced chirality by polarization rotation, measured for different polarization angles of the incident probe. **b**, Polarization rotation signal for uniaxial strain along the *x* direction for different polarization angles of the incident probe. **c**, Rotary power as a function of uniaxial strain, extracted from **b**. **d**, Same as **b** but for uniaxial strain along the *y* direction. **e**, Same as **c** but for uniaxial strain along the *y* direction. Source data

**Extended Data Fig. 2:**
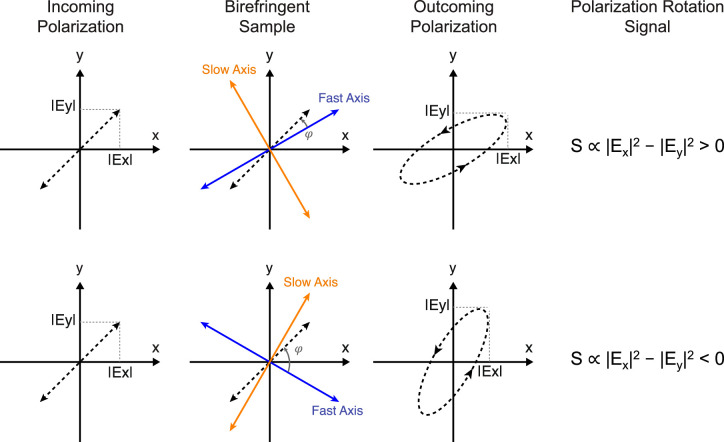
Illustration of the contribution of birefringence to the polarization rotation signal. When linearly polarized light propagates through a birefringent crystal, it becomes elliptically polarized if the incident polarization is not aligned with a principal axis. The resulting imbalance between the detector projections produces a polarization rotation signal. Source data

We experimentally verified the piezochiral effect by measuring the optical activity of a *c*-cut AgGaS_2_ single crystal under strain (Fig. 4a), which directly reflects its geometrical chirality^35,36^. The polarization rotation of a near-infrared probe field, measured over a full 360° range of polarization angles, enabled us to isolate the signal contribution of the optical activity from birefringence effects, which are also induced by the application of strain (Extended Data Fig. 2).

**Extended Data Fig. 3:**
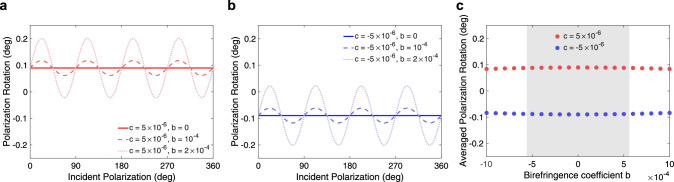
Numerical simulations of polarization rotation in the presence of optical activity and birefringence. **a**, Polarization rotation signal as a function of incident polarization angle for positive optical activity, shown with and without birefringence. **b**, Same as in panel **a** but for optical activity of the opposite sign. **c**, Polarization-averaged rotation signal as a function of birefringence. The shaded region denotes the birefringence range accessed experimentally.

**Extended Data Fig. 4:**
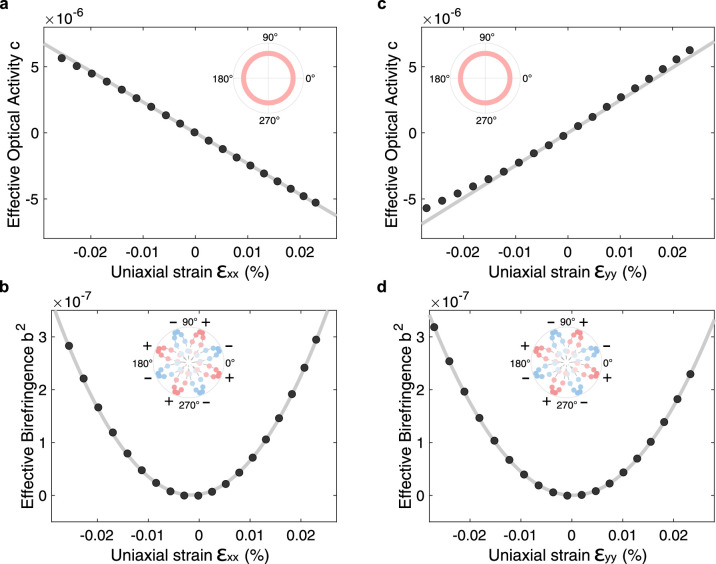
Strain-induced changes in permittivity. **a**, Effective optical activity coefficient *c* as a function of uniaxial strain along the *x* direction. **b**, Square of the effective birefringence coefficient *b*^2^ as a function of uniaxial strain along the *x* direction. **c**, Same as panel **a** as a function of uniaxial strain along the *y* direction. **d**, Same as panel **b** as a function of uniaxial strain along the *y* direction. Source data

Figure 4b shows the results for uniaxial strain applied along the *x* direction. The optical activity is extracted from the data by averaging the polarization rotation signal over the polarizations of the incident probe, which is possible because the strain-induced birefringence signal averages to zero^37,38^. We note that the extraction of optical activity is robust here because AgGaS_2_ exhibits no equilibrium birefringence along the probed direction and the strain-induced birefringence remains a small perturbation, as confirmed by numerical simulations (Extended Data Fig. 3). On the basis of these measurements, together with the parameters listed in Supplementary Table 15, we determine the strain-induced change in the permittivity tensor (Extended Data Fig. 4).

The obtained optical activity shows a linear dependence on the applied strain, providing direct evidence of the piezochiral effect (Fig. 4c). Notably, the handedness of the chiral state is reversed by switching between compressive and tensile strain, highlighting the versatility of the effect in inducing chirality. Further experiments with strain applied along the orthogonal *y* direction show a sign change in the induced optical activity compared with strain along the *x* direction (Fig. 4d,e). Quantitatively, a uniaxial strain of only 0.02% yields a rotary power on the order of 10% of α-quartz^38^, a widely used chiral optical material, demonstrating the large magnitude of the piezochiral response in AgGaS_2_. In combination, these experimental results provide clear verification of strain-induced chirality in AgGaS_2_, with the symmetry in agreement with the theoretically predicted piezochiral effect.

## Summary and outlook

Starting from symmetry considerations, we have introduced the piezochiral effect and established a new formalism to describe the linear coupling between strain and chirality. This effect was verified in the prototype compound AgGaS_2_ through measurements of the strain-induced optical activity. Our discovery expands the family of strain-responsive functionalities by introducing a new member alongside piezoelectricity^28^ and piezomagnetism^29^, thereby opening new possibilities across a broad range of applications. Notably, chirality can be induced by uniform strain within this framework, in contrast to torsional strain, which involves a strain gradient that breaks translational symmetry^39^.

One particularly exciting direction involves the integration of piezochiral materials into microelectromechanical systems (MEMS)^40^, enabling chirality control within compact on-chip platforms. Such integration could lead to the development of enantioselective biosensors^41^, dynamically tunable optical devices and polarization-based information technologies.

Asymmetric catalysis represents another compelling avenue for the piezochiral effect^42,43^. One strategy is to use piezochiral materials directly as asymmetric catalysts, in which strain-controlled chirality could enable rational control over enantioselectivity in specific reactions. Another strategy is to use piezochiral substrates as scaffolds for well-established asymmetric catalysts^44^, providing a means to dynamically tune enantiomeric excess and selectivity, thereby addressing long-standing goals of controllability and versatility in asymmetric synthesis^45^.

Beyond technological applications, the piezochiral effect could serve as a unique channel for coupling strain to emergent chiral order in quantum materials^46–50^, opening opportunities to probe and manipulate new quantum phases. More broadly, the piezochiral effect offers a new framework for chiral matter engineering, with interdisciplinary implications for photonics^51^, spintronics^52–54^, biosensing^55^ and quantum information^56^.

## Methods

### Experimental set-up

**Extended Data Fig. 5:**
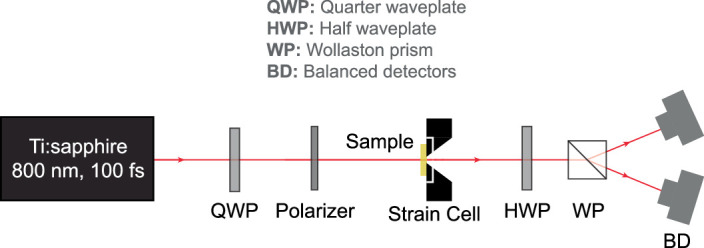
Schematic drawing of the experimental set-up. A narrow band-pass filter (5 nm bandwidth) was used to select a central wavelength of 808 nm from the spectrum of a Ti:sapphire amplifier system. The probe polarization was controlled using a quarter-wave plate and a polarizer and the strain-induced polarization rotation was measured using a combination of a half-wave plate, a Wollaston prism and two photodiodes.

Extended Data Figure 5 shows the schematic of the optical set-up used in the experiment. Probe pulses for the polarization rotation measurements were provided by a femtosecond Ti:sapphire amplifier system and focused onto the sample with a lens to a spot size of approximately 30 µm. A narrow band-pass filter (5 nm bandwidth) was used to select a central wavelength of 808 nm. The probe beam was aligned at normal incidence to the sample surface and its polarization was controlled using a quarter-wave plate and a polarizer. The strain-induced polarization rotation was measured using a combination of a half-wave plate, a Wollaston prism and two photodiodes. Uniaxial strain was applied to the sample using a commercial strain cell (Razorbill Instruments UC220T). The applied strain was determined from the measured stress using the elastic compliance tensor of AgGaS_2_ (Supplementary Table 14). All of the measurements were carried out under ambient conditions.

### Sample preparation

The sample investigated was a commercially available single crystal AgGaS_2_ of 200 µm thickness with an optically flat (001) surface. Strips of 2.5 mm × 0.5 mm were cut from the same crystal along the crystal *a* and *b* axes, respectively, enabling the application of uniaxial strain along both the *x* and *y* directions. These strips were mounted onto separate adapters for the strain cell, enabling exchange of samples during the measurement campaign.

### Wavelength dependence of the optical activity

**Extended Data Fig. 6:**
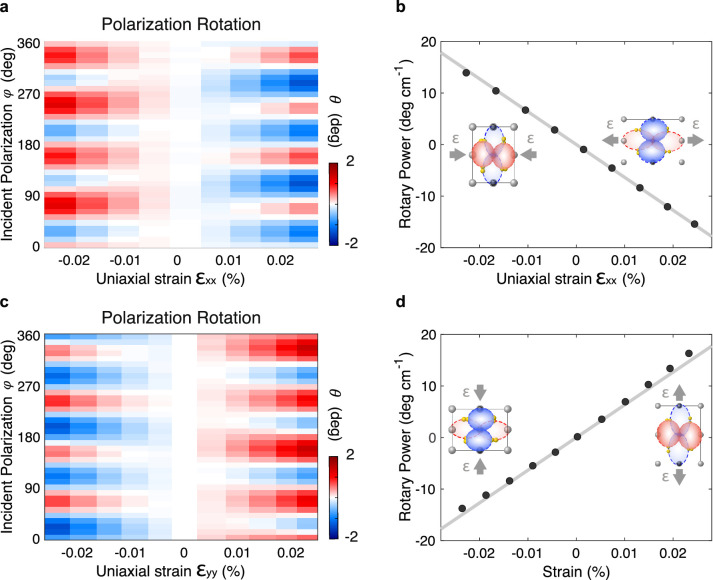
Measurement results at 633 nm probe wavelength. **a**, Polarization rotation signal for uniaxial strain along the *x* direction for different polarization angles of the incident probe. **b**, Rotary power as a function of uniaxial strain, extracted from **a**. **c**, Same as **a** but for uniaxial strain along the *y* direction. **d**, Same as **b** but for uniaxial strain along the *y* direction. Source data

We performed further strain-induced polarization rotation measurements using a second probe wavelength of 633 nm (Extended Data Fig. 6). The probe pulses were generated from the output of the same near-infrared Ti:sapphire amplifier using white-light generation in a sapphire single crystal, followed by isolation of a 1 nm bandwidth with a band-pass filter centred at the target wavelength.

The measurements exhibit strain-induced optical activity with the same qualitative behaviour as observed at 808 nm, however with a much larger rotary power. This behaviour is consistent with the general wavelength dependence in chiral materials, in which the rotary power increases as the probe wavelength approaches the electronic bandgap^31^. In AgGaS_2_, the bandgap energy is approximately 2.76 eV, corresponding to a wavelength of about 450 nm (ref. ^57^).

### Assessment of systematic errors

We performed identical strain-dependent polarization rotation measurements on a centrosymmetric non-piezochiral reference sample, with the goal of assessing the magnitude of potential systematic errors. We chose CaF_2_, with point group $m\bar{3}m$ (Oh) for this purpose.

**Extended Data Fig. 7:**
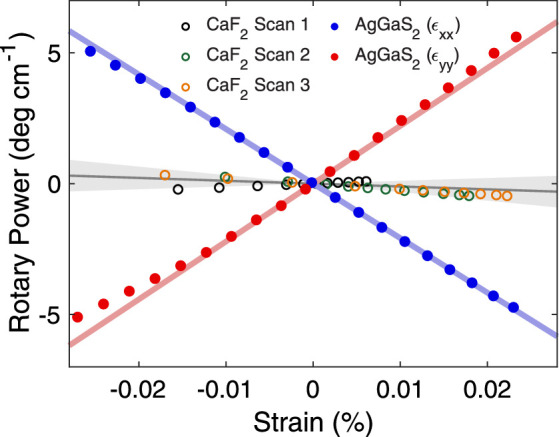
Comparison of strain-induced rotary power in piezochiral AgGaS_2_ and the reference system CaF_2_. The blue and red data represent the optical activity in piezochiral AgGaS_2_ induced by uniaxial strain applied along the crystal *a* and *b* axes, respectively. The black line shows the average linear fit obtained from three independent strain-dependent measurements performed at different positions on the CaF_2_ sample surface. The shaded region represents the standard deviation of these fits. Source data

The CaF_2_ crystal with a (001) surface was prepared with the same thickness as the AgGaS_2_ samples. Uniaxial strain was then applied along the [010] direction and polarization rotation measurements were conducted under identical optical conditions using the 808 nm probe beam. We performed three independent measurements on different positions of the CaF_2_ sample surface (Extended Data Fig. 7). We found a residual strain-induced rotation with amplitude and sign that depend on sample position. These observations are attributed to systematic experimental uncertainties, potentially arising from slight optical misalignment, strain inhomogeneity across the sample or imperfections in the detection optics.

### PiezoChiral Materials Database

We developed an online database for piezochiral materials based on the piezochiral tensor formalism introduced in this work^58^. The database systematically enumerates the piezochiral tensor forms allowed by crystal symmetry and associates them with candidate material systems. Application of this framework yielded a catalogue of more than 40,000 candidate piezochiral materials.

## Online content

Any methods, additional references, Nature Portfolio reporting summaries, source data, extended data, supplementary information, acknowledgements, peer review information; details of author contributions and competing interests; and statements of data and code availability are available at 10.1038/s41586-026-10845-5.

## Supplementary information

Supplementary Information Supplementary sections 1–6 and supplementary references

Peer Review file

## Source data

Source Data Fig. 3

Source Data Fig. 4

Source Data Extended Data Fig. 2

Source Data Extended Data Fig. 4

Source Data Extended Data Fig. 6

Source Data Extended Data Fig. 7

## Data Availability

Source data are provided with this paper. All other data that support the plots in this paper and other findings of this study are available from the corresponding authors.
