# Supplementary material for: The piezochiral effect
Source: Nature. 2026 Jul 29;656(8127):338–42. doi: 10.1038/s41586-026-10845-5 (PMC13468133; doi:10.1038/s41586-026-10845-5)
Supplement: Supplementary file 1 [file 41586_2026_10845_MOESM1_ESM.pdf]

nature portfolio

https://doi.org/10.1038/s41586-026-10845-5

Supplementary information

The piezochiral effect

In the format provided by the authors and unedited

Supplementary Materials for

The Piezochiral Effect

Z. Zeng[1,2], M. Först[1], M. Fechner[1], X. Deng[1,2], A. Cavalleri[1,2], P. G. Radaelli[2]

[1]Max Planck Institute for the Structure and Dynamics of Matter, Hamburg, Germany
[2]Department of Physics, Clarendon Laboratory, University of Oxford, Oxford, United Kingdom

Table of Contents:

S1. Symmetry Analysis of Piezochiral Tensor and Higher-order Piezochiral Tensor

S2. Analysis of Chirality on Local Atomic Sites

S3. Application of Uniaxial Strain

S4. Analysis of the Polarization Rotation Signal

S5. Numerical Simulations of Light Propagation

S6. Chiral Order Parameter Calculations

References

S1. Symmetry Analysis of Piezochiral Tensor and Higher-order Piezochiral Tensor

From a symmetry perspective, chirality is a pseudoscalar quantity with Jahn symbol[1,2] (e) while strain is a 2nd rank symmetric tensor, with Jahn symbol ([V^2]). Therefore, their linear coupling corresponds to a 2nd rank pseudotensor (e[V^2]), which is parity odd and time-reversal even.

For an achiral system, the piezochiral effect can only occur in systems without inversion symmetry, because strain does not break inversion symmetry.

Among the ten point groups fulfilling this prerequisite, only four allow for the linear strain-chirality coupling. Their piezochiral tensor forms are tabulated below in Tables S1-S4.

T_{ij}		i		
		1	2	3
j	1	T_{11}	0	0
	2	0	$-T_{11}$	0
	3	0	0	0

Table S1 | Piezochiral tensor for point group $\bar{4}2m$ (D2d).

T_{ij}		i		
		1	2	3
j	1	0	T_{12}	0
	2	T_{12}	0	0
	3	0	0	0

Table S2 | Piezochiral tensor for point group $mm2$ (C2v).

T_{ij}		i		
		1	2	3
j	1	T_{11}	T_{12}	0
	2	T_{12}	$-T_{11}$	0
	3	0	0	0

Table S3 | Piezochiral tensor for point group $\bar{4}$ (S4).

T_{ij}		i		
		1	2	3
j	1	0	T_{12}	0
	2	T_{12}	0	T_{23}
	3	0	T_{23}	0

Table S4| Piezochiral tensor for point group m (Cs). Here we adopt the convention that the mirror plane is oriented perpendicular to the y-axis.

To explicitly illustrate how chirality emerges in these achiral piezochiral systems, Table S5 shows how specific strain components reduce the parent achiral point groups to chiral subgroups by breaking all improper symmetry operations.

Parent Point Group	Applied Strain Components	Broken Symmetry Operations	Resulting Subgroup
$\bar{4}2m$ (D2d)	$\varepsilon_{xx}/\varepsilon_{yy}$	$\bar{4}^+, \bar{4}^-, m_{110}, m_{1\bar{1}0}$	222 (D2)
$mm2$ (C2v)	ε_{xy}	m_x, m_y	2 (C2)
$\bar{4}$ (S4)	$\varepsilon_{xx}/\varepsilon_{yy}/\varepsilon_{xy}$	$\bar{4}^+, \bar{4}^-$	2 (C2)
m (Cs)	$\varepsilon_{xy}/\varepsilon_{yz}$	m_y	1 (C1)

Table S5 | Strain-induced symmetry lowering. For each parent achiral point group, the strain components that eliminate all improper symmetry operations are listed, along with the resulting chiral subgroup.

By symmetry, the piezochiral coupling is also allowed in chiral systems, where it modulates the chiral order parameter. Below we list the piezochiral tensor forms for the corresponding eleven point groups (Tables S6-S10).

T_{ij}		i		
		1	2	3
j	1	T_{11}	T_{12}	T_{13}
	2	T_{12}	T_{22}	T_{23}
	3	T_{13}	T_{23}	T_{33}

Table S6 | Piezochiral tensor for point group 1 (C1).

T_{ij}		i		
		1	2	3
j	1	T_{11}	0	T_{13}
	2	0	T_{22}	0
	3	T_{13}	0	T_{33}

Table S7 | Piezochiral tensor for point group 2 (C2). Here we adopt the convention that the 2-fold rotation axis is along the y-axis.

T_{ij}		i		
		1	2	3
j	1	T_{11}	0	0
	2	0	T_{22}	0
	3	0	0	T_{33}

Table S8 | Piezochiral tensor for point group 222 (D2).

T_{ij}		i		
		1	2	3
j	1	T_{11}	0	0
	2	0	T_{11}	0
	3	0	0	T_{33}

Table S9 | Piezochiral tensor for point group 4 (C4), 422 (D4), 3 (C3), 32 (D3), 6 (C6) and 622 (D6).

T_{ij}		i		
		1	2	3
j	1	T_{11}	0	0
	2	0	T_{11}	0
	3	0	0	T_{11}

Table S10| Piezochiral tensor for point group 23 (T) and 432 (O).

For the higher-order piezochiral effect, the general coupling term for the nth order writes

$$C = \sum_{i_1 j_1 i_2 j_2 \ldots i_n j_n} T_{i_1 j_1 i_2 j_2 \ldots i_n j_n} \varepsilon_{i_1 j_1} \varepsilon_{i_2 j_2} \ldots \varepsilon_{i_n j_n}$$

where the coupling tensor transforms with the symmetry of e[[V^2]^n].

There are four point groups with the 2nd order piezochiral coupling as the leading term. The corresponding piezochiral coupling forms are summarized in Table S11.

Point group symmetry	Piezochiral coupling
$4mm$ (C4v)	$C = \theta(\varepsilon_{xx} - \varepsilon_{yy})\varepsilon_{xy}$
$3m$ (C3v)	$C = \theta[(\varepsilon_{xx} - \varepsilon_{yy})\varepsilon_{xz} - 2\varepsilon_{xy}\varepsilon_{yz}]$
$\bar{6}m2$ (D3h)	$C = \theta[(\varepsilon_{xx} - \varepsilon_{yy})\varepsilon_{xz} - 2\varepsilon_{xy}\varepsilon_{yz}]$
$\bar{6}$ (C3h)	$C = \theta_1[(\varepsilon_{xx} - \varepsilon_{yy})\varepsilon_{xz} - 2\varepsilon_{xy}\varepsilon_{yz}] + \theta_2[(\varepsilon_{xx} - \varepsilon_{yy})\varepsilon_{yz} + 2\varepsilon_{xy}\varepsilon_{xz}]$

Table S11 | Second-order piezochiral effect. Point group symmetries and their corresponding piezochiral coupling forms.

There are also two groups with the 3rd order piezochiral coupling as the leading term. The corresponding piezochiral coupling forms are summarized in Table S12.

Point group symmetry	Piezochiral coupling
$\bar{4}3m$ (Td)	$C = \theta_1(\varepsilon_{xx} - \varepsilon_{yy})(\varepsilon_{yy} - \varepsilon_{zz})(\varepsilon_{zz} - \varepsilon_{xx}) + \theta_2[(\varepsilon_{xx} - \varepsilon_{yy})\varepsilon_{xy}^2 + (\varepsilon_{yy} - \varepsilon_{zz})\varepsilon_{yz}^2 + (\varepsilon_{zz} - \varepsilon_{xx})\varepsilon_{xz}^2]$
$6mm$ (C6v)	$C = \theta_1[\varepsilon_{xy}(\varepsilon_{yz}^2 - \varepsilon_{xz}^2) + \varepsilon_{xz}\varepsilon_{yz}(\varepsilon_{xx} - \varepsilon_{yy})] + \theta_2[\varepsilon_{xy}(3\varepsilon_{xx}^2 + 3\varepsilon_{yy}^2 - 6\varepsilon_{xx}\varepsilon_{yy} - 4\varepsilon_{xy}^2)]$

Table S12 | Third-order piezochiral effect. Point group symmetries and their corresponding piezochiral coupling forms.

In total, all the ten achiral non-centrosymmetric point groups allow for the piezochiral coupling, with the lowest-order contribution being first order for four groups, second order for four groups, and third order for two groups. The systematic classification is summarized in Extended Data Figure 1.

In practice, these higher-order effects can be accessed by applying specific combinations of strain components. For instance, tensile strain applied along the [111] body diagonal in cubic GaAs (point group $\bar{4}3m$) generates equal strain in ε_{xx}, ε_{yy} and ε_{zz}, which, according to the symmetry-allowed coupling form, leads to the emergence of chirality.

S2. Analysis of Chirality on Local Atomic Sites

To assess the generality of the proposed mechanism of piezochirality, we analyze the presence of local chiral environments at the level of individual atomic sites. The analysis is performed for the piezochiral crystal systems listed in the manuscript, and is based on the space group symmetries and corresponding Wyckoff positions of each structure in the unstrained state. An atomic site is considered locally chiral if its site symmetry lacks improper symmetry operations. In such cases, a chiral order parameter can be defined in terms of electric toroidal monopole[3,4]. The results of this analysis are summarized in Table S13. For all crystal systems considered, at least one atomic site resides in a locally chiral environment. This demonstrates that the presence of local chirality is a common feature among materials in which piezochiral coupling is symmetry-allowed. Since these systems are globally achiral in the absence of external perturbations, the local chiral environments are compensated by symmetry-related counterparts of opposite handedness, resulting in an antiferrochiral arrangement.

Strain application partially lifts the symmetry operations that relate these opposite-handed local environments, rendering them inequivalent, and enabling the emergence of a net chiral response at the macroscopic level. The analysis supports the general picture that piezochiral effects can arise from the imbalance of compensated local chiral substructures in a broad class of materials.

Crystal system	Point group	Space group	Chiral atomic site
$AgGaS_2$	-42m	I-42d	S(8d)
γ-$LiBO_2$	-42m	I-42d	O(8d)
$KH2PO_4$	-42m	I-42d	H(8d), O(16e)
$ZnGeP_2$	-42m	I-42d	P(8d)
LiB_3O_5	mm2	$Pna2_1$	Li(4a), B(4a), O(4a)
$KTiPO_5$	mm2	$Pna2_1$	K(4a), Ti(4a), P(4a), O(4a)
Bi_2SeO_5	mm2	Aem2	O(4b), Bi(8d), Se(8d), O(8d)
SrB_4O_7	mm2	$Pmn2_1$	B(4b), O(4b)
BPO_4	-4	I-4	O(8g)
$CdAl_2S_4$	-4	I-4	S(8g)
NaI_3O_8	-4	P-4	I(2g), I(4h), O(4h)
$CsAs_4F_{13}$	-4	I-4	As(8g), F(8g)
$Cd(AlCl_4)_2$	m	Pc	Cd(2a), Al(2a), Cl(2a)
$SnTa_2O_8$	m	Cc	Sn(4a), Ta(4a), O(4a)
$Te_2P_2O_9$	m	Cc	Te(4a), P(4a), O(4a)
$As_2O(SO_4)_2$	m	Pc	As(2a), O(2a), S(2a)

Table S13 | Chiral atomic sites in the piezochiral crystal systems. All atomic sites with local chiral environments are listed together with their corresponding Wyckoff positions. The same chemical element appearing at different Wyckoff positions denotes symmetry-inequivalent sites of that element within the crystal. The analysis is performed for the unstrained state.

S3. Application of Uniaxial Strain

The strain cell employs piezo-stack actuators to generate a tunable uniaxial stress, both compressive and tensile. It is equipped with a sensor that monitors the force applied to the sample. The applied stress σ is evaluated from the ratio between the measured force and the cross-section area of the sample. The resulting strain ε is then calculated from the stress using the compliance tensor S of the crystal[5]

$$\varepsilon_{ij} = S_{ijkl}\sigma_{kl}$$

For uniaxial stress applied along the x direction (σ_{xx}), the crystal symmetry ($\bar{4}2m$) ensures that all off-diagonal shear strain components are zero. The nonzero strain components are expressed as follows, with the corresponding compliance constants S_i listed in Table S14.

$$\begin{aligned} \varepsilon_{xx} &= S_1\sigma_{xx} \\ \varepsilon_{yy} &= S_2\sigma_{xx} \\ \varepsilon_{zz} &= S_3\sigma_{xx} \end{aligned}$$

For uniaxial stress applied along the orthogonal y direction (σ_{yy}), the relations are

$$\begin{aligned} \varepsilon_{yy} &= S_1\sigma_{yy} \\ \varepsilon_{xx} &= S_2\sigma_{yy} \\ \varepsilon_{zz} &= S_3\sigma_{yy} \end{aligned}$$

Compliance Constant	Value (10^{-12} Pa^{-1})
$\boldsymbol{S_1}$	26.2
$\boldsymbol{S_2}$	-7.7
$\boldsymbol{S_3}$	-14.5

Table S14. | Relevant compliance constants of $AgGaS_2$[5].

S4. Analysis of the Polarization Rotation Signal

In the experiments, the probe pulses propagate along the optical axis (*c*-axis) of the crystal, with polarization in the *a*-*b* plane. Hence, only the in-plane permittivity tensor elements are relevant and considered as follows.

For unstrained $AgGaS_2$ with $\bar{4}2m$ (D2d) symmetry, the in-plane permittivity is isotropic:

$$\boldsymbol{\varepsilon} = \begin{pmatrix} \varepsilon_{11} & 0 \\ 0 & \varepsilon_{22} \end{pmatrix} = \begin{pmatrix} \varepsilon_{11} & 0 \\ 0 & \varepsilon_{11} \end{pmatrix}.$$

Uniaxial strain along the crystal *a* or *b* axis reduces the symmetry of the system to a chiral point group 222 (D2), inducing both birefringence and optical activity. The birefringence modifies the diagonal elements of the permittivity tensor

$$\boldsymbol{\varepsilon}' = \begin{pmatrix} \varepsilon_{11} - b & 0 \\ 0 & \varepsilon_{11} + b \end{pmatrix},$$

while the strain-induced optical activity (g_{33}) introduces antisymmetric imaginary off-diagonal terms following

$$\Delta\varepsilon_{ij} = \boldsymbol{i}\, l_{ijk} g_{kl} k_l$$

where l_{ijk} is the Levi-Civita symbol and k is the wavevector.

Combining both effects, the permittivity tensor is given by

$$\boldsymbol{\varepsilon}'' = \begin{pmatrix} \varepsilon_{11} - b & ic \\ -ic & \varepsilon_{11} + b \end{pmatrix},$$

with

$$c = g_{33} k_3$$

the effective optical activity and b the effective birefringence.

We note that for unstrained $AgGaS_2$ with $\bar{4}2m$ (D2d) symmetry, the optical activity tensor is diagonal, with $g_{11} = -g_{22}$ and $g_{33} = 0$. Since the probe pulses propagate along the optical axis (*c*-axis) of the crystal, only the g_{33} component contributes to the polarization rotation signal and directly reflects the presence of chirality induced by strain.

The incident linearly polarized probe pulse can be decomposed into two eigenvectors following the permittivity tensor in the strained state described above. Defining $f = \frac{c}{b}$, the eigenvectors in the *a*-*b* plane are two elliptically polarized light waves with opposite ellipticity[6].

$$\boldsymbol{e_1} = \begin{pmatrix} \dfrac{i(-1+\sqrt{1+f^2})}{f\sqrt{1+\frac{(-1+\sqrt{1+f^2})^2}{f^2}}} \\ \dfrac{1}{\sqrt{1+\frac{(-1+\sqrt{1+f^2})^2}{f^2}}} \end{pmatrix} \qquad \boldsymbol{e_2} = \begin{pmatrix} \dfrac{-i(1+\sqrt{1+f^2})}{f\sqrt{1+\frac{(1+\sqrt{1+f^2})^2}{f^2}}} \\ \dfrac{1}{\sqrt{1+\frac{(1+\sqrt{1+f^2})^2}{f^2}}} \end{pmatrix}$$

The effective refractive indices for the two eigenvectors are

$$n_1 = \sqrt{\varepsilon_{11} + \sqrt{b^2 + c^2}} \approx \sqrt{\varepsilon_{11}} + \frac{\sqrt{b^2 + c^2}}{2\sqrt{\varepsilon_{11}}}$$

$$n_2 = \sqrt{\varepsilon_{11} - \sqrt{b^2 + c^2}} \approx \sqrt{\varepsilon_{11}} - \frac{\sqrt{b^2 + c^2}}{2\sqrt{\varepsilon_{11}}}.$$

These approximations hold under the conditions $|b| \ll \varepsilon_{11}$ and $|c| \ll \varepsilon_{11}$.

The phase delay γ between the two eigenvectors is the product of the sample thickness d and the difference in refractive index.

$$\gamma = \frac{2\pi d}{\lambda_0}(n_1 - n_2) \approx \frac{2\pi d}{\lambda_0}\frac{\sqrt{b^2 + c^2}}{\sqrt{\varepsilon_{11}}}$$

The two eigenvectors are then recombined after passing through the sample. The polarization rotation signal is analyzed with a half waveplate and Wollaston prism.

Using the Jones matrix analysis[7], the effective polarization rotation θ is proportional to the normalized intensity difference on the two detectors following

$$\theta(\varphi) = \frac{I_1 - I_2}{2(I_1 + I_2)} = \frac{1}{2\sqrt{1 + 1/f^2}}\sin(\gamma) + \frac{(\cos(\gamma) - 1)}{4(1 + f^2)}\sin(4\varphi)\,.$$

Here, φ is the incident polarization angle relative to the strain axis. Expanding $\sin(\gamma) \approx \gamma$ and $\cos(\gamma) \approx 1 - \frac{\gamma^2}{2}$, we arrive at

$$\theta(\varphi) = \frac{\pi d}{\lambda_0\sqrt{\varepsilon_{11}}}c - \frac{\pi^2 d^2}{2{\lambda_0}^2\varepsilon_{11}}\sin(4\varphi)b^2\,.$$

It is clear from this expression that the signal is composed of two contributions. The first one is proportional to the optical activity coefficient c and does not depend on the incident probe polarization. The second contribution is proportional to the square of the birefringence coefficient, b^2, and depends on the incident probe polarization φ with a 90°-periodicity, consistent with the experimental observations. Importantly, the birefringence contribution averages to zero when integrated over all incident probe polarizations, allowing us to extract the optical activity from measurements of the polarization rotation for all the 360° incident probe polarization angles.

The strain-induced change in the permittivity tensor (Extended Data Fig. 4) can thus be acquired based on the experimental results and the parameters listed in Table S15. Both the effective birefringence b and optical activity c are much smaller than $\boldsymbol{\varepsilon_{11}}$, validating the approximations used in the analysis above.

Physical Quantity	Value
Sample thickness $\boldsymbol{d}$	200 µm
Probe wavelength $\boldsymbol{\lambda_0}$	808 nm
Static permittivity[8] $\boldsymbol{\varepsilon_{11}}$	6.19

Table S15 | Relevant experimental parameters.

Finally, the rotary power ρ is obtained as

$$\rho = \frac{\pi c}{\lambda_0 \sqrt{\varepsilon_{11}}}.$$

Extended Data Figure 2 provides an intuitive illustration of the birefringence contribution to the polarization rotation signal. When a linearly polarized pulse passes through a birefringent crystal, the transmitted beam becomes elliptically polarized if the incoming polarization is not aligned with one of the principal axes. The projection on the x- and y-axes, the balancing axes for the incoming polarization, differ depending on the relative angle between the incoming polarization and the fast axis of the birefringent material. The resulting signal exhibits a four-fold symmetry with respect to the incoming polarization.

S5. Numerical Light Propagation Simulations

We performed numerical simulations of light propagation in a piezochiral material based on the formalism described above. In detail, we decomposed the incident linearly polarized light into the elliptically polarized eigenmodes of the strained crystal, propagated these modes through the sample, recombined them at the output, and calculated the resulting polarization rotation signal.

The simulations were carried out using the parameters corresponding to the experimental conditions listed in Table S15. The calculated polarization rotation signal as a function of incident polarization angle for different strengths of birefringence and optical activity are presented in Extended Data Figure 3.

In the absence of birefringence, the polarization rotation signal does not depend on the incident polarization angle. Already weak birefringence generates the four-fold modulation, however the polarization-averaged signal remains largely unchanged. This behavior is observed for both signs of the optical activity. Within the range of birefringence observed experimentally ($<1\times10^{-3}$), its effect on the polarization-averaged signal, arising from the optical activity, is negligible.

These simulations confirm that, in the experimental regime of the present study, averaging over the incident probe polarization provides an accurate measure of the strain-induced optical activity.

S6. Chiral Order Parameter Calculations

We performed first-principles calculations in the framework of density functional theory (DFT) to explore the impact of strain on the structure of $AgGaS_2$. Our computations are carried out with the Vienna *Ab initio* Simulation Package (VASP, version 6.5.1)[9-11], using pseudopotentials generated within the Projector Augmented Wave (PAW) method[12]. To parametrize the exchange-correlation energy, we applied the PBEsol[13] approximation and used the default potentials of the VASP package for Ga, Ag, and S.

For the numerical settings, we sampled the reciprocal space with an 8×8×4 k-point mesh generated using the scheme of Monkhorst and Pack[14]. We also set the plane-wave cutoff to 550 eV. Both settings were applied consistently to all our computations. Additionally, for all calculations, we iterated the self-consistent cycle until the change in total energy was smaller than 10^{-8} eV.

The starting point of our investigation is the structural relaxation of the $AgGaS_2$ unit cell. Within PBEsol, we find the equilibrium volume to be 339.43 Å^3 (a = b = 5.66 Å, c = 10.55 Å). The atomic equilibrium positions were obtained at the following Wyckoff sites: Ga (4a), Ag (4b), and S (8d, x = 0.77944). Using this relaxed structure, we performed additional structural relaxations under the constraint of a fixed, modulated a- or b-axis. This modulation breaks the tetragonal symmetry, as discussed above, and induces a net chiral state. The resulting atomic positions were then used to compute the chiral order parameter according to Eq. (4) and as shown in Fig. 3 of the main manuscript.

References

1 Jahn, H. Note on the Bhagavantam–Suranarayana method of enumerating the physical constants of crystals. *Acta Crystallographica* **2**, 30-33 (1949).

2 Gallego, S. V., Etxebarria, J., Elcoro, L., Tasci, E. S. & Perez-Mato, J. M. Automatic calculation of symmetry-adapted tensors in magnetic and non-magnetic materials: a new tool of the Bilbao Crystallographic Server. *Foundations of Crystallography* **75**, 438-447 (2019).

3 Oiwa, R. & Kusunose, H. Rotation, electric-field responses, and absolute enantioselection in chiral crystals. *Physical Review Letters* **129**, 116401 (2022).

4 Inda, A., Oiwa, R., Hayami, S., Yamamoto, H. M. & Kusunose, H. Quantification of chirality based on electric toroidal monopole. *The Journal of Chemical Physics* **160** (2024).

5 Grimsditch, M. & Holah, G. Brillouin scattering and elastic moduli of silver thiogallate (AgGaS2). *Physical Review B* **12**, 4377 (1975).

6 Woodford, S., Bringer, A. & Blügel, S. Interpreting magnetization from Faraday rotation in birefringent magnetic media. *Journal of applied physics* **101** (2007).

7 Jones, R. C. A new calculus for the treatment of optical systems I. Description and discussion of the calculus. *JOSA* **31**, 488-493 (1941).

8 Takaoka, E. & Kato, K. Thermo-optic dispersion formula for AgGaS2. *Applied Optics* **38**, 4577-4580 (1999).

9 Kresse, G. & Hafner, J. Ab initio molecular dynamics for liquid metals. *Physical Review B* **47**, 558 (1993).

10 Kresse, G. & Furthmüller, J. Efficiency of ab-initio total energy calculations for metals and semiconductors using a plane-wave basis set. *Computational Materials Science* **6**, 15-50 (1996).

11 Kresse, G. & Furthmüller, J. Efficient iterative schemes for ab initio total-energy calculations using a plane-wave basis set. *Physical Review B* **54**, 11169 (1996).

12 Kresse, G. & Joubert, D. From ultrasoft pseudopotentials to the projector augmented-wave method. *Physical Review B* **59**, 1758 (1999).

13 Perdew, J. P. *et al.* Restoring the density-gradient expansion for exchange in solids and surfaces. *Physical Review Letters* **100**, 136406 (2008).

14 Monkhorst, H. J. & Pack, J. D. Special points for Brillouin-zone integrations. *Physical Review B* **13**, 5188 (1976).
