# Supplementary material for: The piezochiral effect
Source: Nature. 2026 Jul 29;656(8127):338–42. doi: 10.1038/s41586-026-10845-5 (PMC13468133; doi:10.1038/s41586-026-10845-5)
Supplement: Supplementary file 2 [file 41586_2026_10845_MOESM2_ESM.pdf]

nature portfolio

Peer Review File

The Piezochiral Effect

Corresponding Author: Professor Andrea Cavalleri

Any redactions in this file are there to maintain patient confidentiality, the confidentiality of unpublished data, or to remove third-party material.

Parts of this Peer Review File have been redacted as indicated to remove third-party material.

Attachments originally included by the reviewers as part of their assessment can be found at the end of this file.

Version 0:

Reviewer comments:

Referee #1

(Remarks to the Author)
The manuscript claims that mechanical strain can induce chirality in a solid that is otherwise achiral. This is an appealing idea, and strain engineering is indeed an important tuning approach in condensed matter physics. However, in its current form the paper does not yet meet the standards required for Nature. The evidence for a genuine chirality transition is not convincingly established, and the manuscript does not sufficiently address relevant existing literature where similar effects have already been investigated.

The novelty of the work is not clearly supported by the literature discussion. There already exist studies where external fields drive a transition from an achiral state to a chiral one. In particular, electric field control of chirality in achiral ferroaxial $NiTiO_3$ has been demonstrated. (Phys. Rev. Mater. 5, 124409 (2021)). Strain stabilized chiral phases have been reported in $BaTiS_3$ (Adv. Funct. Mater. 35, 2416560 (2025)). Pressure and strain tunability of achiral to chiral transitions has been analyzed for K_3NiO_2 (Phys. Rev. B 111, 174102 (2025)). A systematic framework for such displacive chiral transitions has also been developed recently (arXiv 2503.13076v1). These works must be cited and the authors should clarify what new principle is established in the present study that goes beyond them.

An issue concerns the symmetry analysis. To demonstrate that chirality has emerged, it must be shown explicitly that all improper symmetry operations such as mirror and inversion are removed by the applied strain. The manuscript states that the symmetry changes to a chiral point group but does not specify which symmetry elements are broken in each strain configuration. Without a clear and complete account of the symmetry lowering process, the optical rotation data may reflect only strain induced anisotropy or local distortions rather than true crystallographic chirality.

Experimentally, there is no demonstration of reproducibility, no temperature or wavelength dependence, and no discussion of possible systematic effects like strain induced birefringence. For claims of this importance, validation through multiple independent measurements is needed.

The statistical reporting is insufficient. Error bars are not defined in the figures, the number of repeated measurements is unclear, and no quantitative assessment of uncertainty or significance is presented. Given the small magnitude of the reported rotation, this is a substantial weakness.

In summary, the direction is interesting and potentially important, but major revisions are needed before this manuscript could be considered for publication in Nature. Explicit differentiation from existing studies including those cited above, more rigorous symmetry analysis, and significantly strengthened experimental evidence would be required to support the central claims.

Referee #2

(Remarks to the Author)
In this article, the authors develop on, and prove the existence of a new crystal property coined piezochiral effect, which

refers to the induction and control of chirality in achiral crystalline materials through strain (similarly to piezoelectricity for polarization).
After a definition of the piezochiral tensor, they first analyse which crystal point groups can host this effect through a formal symmetry and group theory approach. The authors demonstrate that uniaxial strain can induce chirality in materials with specific crystallographic point groups that they list. From this list, they could identify the model system AgGaS2 (point group -42m) where they show the existence of this piezochiral response through both experiments and DFT calculations. Both clearly show the presence of a linear evolution of chirality amplitude versus uniaxial strain amplitude in AgGaS2, which means that chirality change sign with the sign of the strain (i.e. compressive or tensile). From the simulation side, by defining a chiral order parameter (a pseudoscalar function depending on atomic positions that reflects handedness) to quantify chirality and check how it evolves versus uniaxial strain in AgGaS2. From the experimental side, they do so by measuring the rotatory power of a linearly polarized light through the sample with respect to uniaxial strain.
As both, theory and experiment, confirm the same linear evolution of the induced chirality versus uniaxial strain in AgGaS2, it gives a very strong confirmation and validation of the existence of the piezochiral effect.
The authors also discuss higher-order piezochiral couplings and the integration of piezochiral materials into microelectromechanical systems (MEMS) for on-chip chirality manipulation.
The discovery clearly opens new avenues for chirality control in solid-state systems, with potential applications in spintronics, asymmetric catalysis, biosensing, or quantum materials.

The paper is clearly written, it is sufficiently well popularized for a boarder audience of non-specialists, it introduces a new concept never observed or proved before, it opens new avenues as this is quite novel with broad interdisciplinary implications, and it combines carefully done symmetry analysis, first-principles simulations, and experimental validation such that I think it checks all the points to be published in Nature journal.

I have the following comments that should be responded by the authors before acceptance, but I think they are quite easy to respond:

1) The authors demonstrate the presence of a piezochiral response in achiral space groups. However, why not in chiral space groups? Intuitively, one would expect a piezochiral response in chiral space groups as well, analogous to how piezoelectricity exists in polar systems, or did I missed something on the way of why a focus is done on achiral systems? Addressing this could further generalize the scope and impact of the paper.

2) It is, in a way, related to my comment 1, but I think there are already some previous works playing with strain to tune chirality in chiral crystals, for example in PRB 102, 235127 (2020) or with pressure (which is in the way the converse effect of strain) in J. Phys. Soc. Jpn. 43, pp. 195-203 (1977). I think the authors should extend their introduction regarding previous works that played with strain or stress and chirality or related properties, even if this was not called “piezochirality” it is nevertheless very close/related to the present work.

3) For the symmetry analysis, I would have liked to also see what are the symmetry that are broken in each point groups and what are the associated subgroup, could the authors add such information if they also think it can be useful (probably in SI as it would be of interest to more specialist than board audience?)?

4) Upon carefully reviewing the literature to determine whether the term piezochirality is indeed novel, we, however, can find such a mention in J. Phys.: Condens. Matter 37, 163004 (2025). In that article, the term “piezochirality” was anticipated as a potential crystal response, but no experimental nor computational nor symmetry evidence was provided to confirm its existence as done here. This prior mention does not reduce the novelty and impact of the present study, which rigorously establishes the effect through symmetry analysis, DFT calculations, and experimental validation. However, for completeness and fairness, I think it would be appropriate to acknowledge this anticipation in the discussion.

5) I found this typo in the bibliography: Refence 19, “mangamse” should be “manganese”

6) Why is there two references lists in the SI with the same numbering? This is confusing, the authors should clean it.

So, beside a careful check of previous literature (i.e. put in better perspective previous works w.r.t. the present work) as a major comment, I think the paper fit very well with Nature publication rules.

Referee #3

(Remarks to the Author)
The authors of this work show how the application of strain deletes all the mirror planes of a material, hence introducing a chiral state. Its sign can be selected through the sign or the direction of the strain, and its presence is demonstrated in strain-dependent measurements of the optical activity. The relation between strain and chirality is denominated as piezochiral effect.

The key point of this manuscript is to show that chirality in a material can be generated and switched in a controlled manner. This is a fundamental and straightforward idea, and apart from the points listed below, its derivation and demonstration are very convincing. Likewise, introduction of the piezochiral effect is fundamental and clear. Even if I have some concerns about novelty and generality, I would support publication if the following points can be mended.

(1) There is a larger context than the one given by the authors. There are quite a number of examples in the literature where the controlled creation of chiral states has been discussed. The authors mention Refs. 11-13. Other examples where the topic is covered (there are more!) are found in Phys. Rev. B 111, 174102 (2025) (K_3NiO_2 and related oxides; see Refs 28, 29 in this one), Chem. Eur. J. 23, 7990 (2017) (metal-organic frameworks), Science 384, 1096 (2024) (nematic systems), Microscopy and Microanalysis 30, 1409 (2024) (quasi-1D $BaTiS_3$). A review in Materials 15, 5812 (2022) discusses how continuous structural parameters (e.g., internal coordinate u) can be tuned, including by external pressure, to move smoothly between achiral and chiral configurations. I agree that all these are different from the present work, and the piezochiral behavior is not discussed. Nevertheless the authors should put their contribution in relation to this considerable body of work.

(2) I cannot verify Eq. (2) as the specific form of Eq. (1) for -42m symmetry. In that point-group symmetry, group-theoratical tables show that we have T_yy = Txx and epsilon_yy = -epsilon_xx. Therefore, C = T_xx epsilon_xx + T_yy epsilon_yy = 0. In particular, the distinction between epsilon_xx and epsilon_yy made in Eq. (2) does not make sense to me because of the negative equality of the two.

(3) Little is said about the DFT calculations. For a verification of the chiral degeneracy, it is not needed: here the discussion of symmetry is sufficient. DFT produces the order of magnitude of the effect, but aside from a reference to Fig. 3 it is not discussed.

(4) The term “incident polarization” is repeatedly used. Polarization is not incident, however -- light is.

(5) I find Fig. 2 confusing. I do not see how the atoms in panels a, b, c correspond to one another, and I do not see the antichiral relation between the upper and the lower half of the unit cell in panel a. What exactly do the lobes in panel c represent (what are the dashed lines?), and how does this correspond to panels a and b?

(5) To me the conclusions appear as exaggerated. The effect demonstrated here is fundamentally important, but the effect is still rather special, and I do not see that broad spectrum of applications up to MEMS and on-chip applications. How would strain control be exerted on a chip? Also, the magnitude of the reported effect is quite small. For the applications discussed in lines 127-137 I do not see the need for strain-controlled chirality. To my impression, an intrinsically chiral constituent may be used just as well.

Version 1:

Reviewer comments:

Referee #1

(Remarks to the Author)
The manuscript proposes that mechanical strain can induce and control chirality in crystalline solids and introduces the concept of a piezochiral effect. The idea is appealing, and the combination of symmetry analysis, first-principles calculations, and optical measurements is potentially suitable for a broad audience. However, despite the authors' revisions and detailed response, I remain unconvinced that the central claims are established with the level of rigor required for publication in Nature.

My main concern relates to the identification of optical activity in the experiment. The authors interpret a polarization-independent component of the measured rotation signal as evidence for strain-induced chirality, arguing that birefringence contributions can be removed by averaging over the incident polarization angle. While this argument is formally valid under ideal conditions, it is not sufficient in practice. A polarization-independent offset is not uniquely indicative of optical activity and may arise from residual misalignment, imperfections in the polarization optics, or other systematic effects. The reference measurements on a centrosymmetric material show finite strain-dependent rotation signals, even though the effect should be forbidden by symmetry. The presence of such background signals raises concerns about whether the observed response in the target material can be unambiguously attributed to intrinsic optical activity. Moreover, no direct measurement of circular birefringence or related quantities is provided, so the identification of chirality remains indirect.

A second concern is the claimed generality of the proposed mechanism. The authors argue that the piezochiral effect applies broadly to non-centrosymmetric materials. However, the specific mechanism demonstrated relies on the presence of compensated chiral substructures of opposite handedness within the unit cell, whose degeneracy is lifted by strain. It is not clear that such arrangements are common among non-centrosymmetric crystals. In many materials, chirality is either absent or already fixed globally, and there is no obvious internal degree of freedom that can be unbalanced in this way. While a coupling between strain and chirality may be symmetry-allowed in a formal sense, the physical realization illustrated here appears to be significantly more restricted than suggested.

In summary, although the direction of the work is interesting, the experimental evidence for strain-induced optical activity is not yet sufficiently compelling, and the scope of the proposed effect is overstated. I therefore do not consider the manuscript suitable for publication in Nature in its present form.

Referee #2

(Remarks to the Author)
The authors have addressed all of my comments but one, i.e. a minor discrepancy regarding a promised acknowledgment that should be rectified before final acceptance.

Below is a detailed response:

- Focus on achiral systems: The authors provided a clear justification for focusing on achiral systems, noting that these materials allow for the deterministic switching between handedness by changing the direction or sign of applied strain.

- As requested, the authors have included a detailed symmetry analysis in the revised Supplementary Information, explicitly listing the broken symmetry operations and the resulting chiral subgroups for various strain configurations and through a systematic classification of the piezochiral coupling for all point groups in the Supplementary Information, this is very good.

- Literature integration: The authors included the relevant earlier works I mentioned (PRB 102, 235127 (2020) and J. Phys. Soc. Jpn. 43, 195-203 (1977)).
However, in their rebuttal, the authors agreed to acknowledge that the term "piezochirality" was anticipated in a recent review (J. Phys.: Condens. Matter 37, 163004 (2025)). While they have added this paper as Reference 19, the current manuscript text uses the citation only to categorize the prior work as a limited existing approach that merely modulates"chirality, but this does not sounds to be associated to the anticipation I was mentionning. To fulfill their rebuttal promise, the authors should revise the wording at this citation to explicitly credit the conceptual anticipation of the term.

- In the limit of my understanding, the authors addressed the other referees' concerns by giving rigorous statistical validation through multi-wavelength and control experiments, performing a detailed symmetry analysis of the piezochiral pseudo-tensor, and clearly distinguishing their work from prior literature as a unique method for the deterministic selection of handedness.

The combination of rigorous symmetry analysis, first-principles simulations, and experimental validation in $AgGaS_2$ remains a very strong confirmation of the piezochiral effect.
I recommend the manuscript for acceptance, providing the minor change with Reference 19 to accurately reflect the authors' rebuttal commitment regarding the anticipation of the "piezochirality" concept.

Referee #3

(Remarks to the Author)
After reading the comments of all the reviewers and the authors' convincing and instructive reply to them, I am now happy to recommend the manuscript for publication. There is one final point, the authors may like to take into account because it remains slightly confusing.

When considering the chirality C = T(ij) epsilon(ij), then the use of group-theoretical tables in R. R. Birss, Symmetry and Magnetism, North-Holland 1966, reveals that C and T(ij) are axial i-tensors of even rank (0 and 2, respectively), whereas epsilon(ij) is a polar i-tensor of even rank (2). For the point group -42m we get for these (tables 7, 4b, 4d in Birss): C = 0, T(xx) = -T(yy), epsilon(xx) = epsilon(yy) and epsilon(zz). All other components are 0. In other words, the point group does not allow any chirality. The point is that the applied stress reduces the symmetry to a subgroup of -42m where the effect is allowed. This is exactly the point of this work. However, with this, statements like "in the point group -42m, the piezochiral coupling takes the form…" are misleading because once we have the strain, we are no longer in -42m. This confused me in my original report, and since the symmetry breaking is the key of the work, I suggest that the authors use more careful wording here.

Version 2:

Reviewer comments:

Referee #1

(Remarks to the Author)
The manuscript presents an interesting and potentially important idea. In particular, I find the central symmetry argument physically appealing. The proposed mechanism, in which strain lifts the compensation between opposite local chiral motifs and induces a net chiral response, appears conceptually general and worthy of broad attention. In my view, this mechanism is closely analogous to the emergence of a net ferromagnetic component when an external magnetic field perturbs a compensated antiferromagnetic state. This gives the work a clear conceptual significance beyond the specific material studied here.

At the same time, I remain concerned that the optical analysis does not yet reach the level of rigor that I would expect for publication in Nature.

My main concern is not whether the observed signal is intrinsic in a broad phenomenological sense. The strain sign reversal, the orthogonal strain dependence, and the wavelength reproducibility together make a convincing case that the effect is not a trivial experimental artifact. Rather, my concern is more specific: whether the measured polarization rotation can be unambiguously interpreted as a direct measure of strain-induced optical activity itself.

The authors correctly point out in the rebuttal that, in the strained state, linear birefringence and gyrotropic effects coexist and the optical eigenmodes become elliptically polarized. However, once this is acknowledged, the interpretation of the measured rotation angle becomes substantially more subtle. In such a regime, the observed polarization evolution is generally not equivalent to a direct measurement of circular birefringence or optical activity. Instead, it reflects a coupled propagation problem involving birefringence, gyrotropic coupling, phase accumulation, sample thickness, and input polarization conditions.

This issue has been analyzed carefully in the literature. In particular, Woodford et al. demonstrated that, in birefringent gyrotropic media, the observed polarization rotation can become a highly nontrivial function of propagation effects even in ideal optical systems where ordinary alignment artifacts are absent [S. R. Woodford, A. Bringer, and S. Blugel, J. Appl. Phys. 101, 053912 (2007)]. The important point is that these complications arise intrinsically from electromagnetic wave propagation itself, rather than from experimental imperfections.

By contrast, the present manuscript relies primarily on perturbative extraction procedures and angular averaging arguments. While these methods are reasonable as phenomenological analysis tools, they do not rigorously establish that the extracted quantity is uniquely proportional to the intrinsic gyrotropic tensor component. In particular, the rebuttal convincingly argues that ordinary birefringence artifacts are unlikely to dominate the signal, but this is not equivalent to demonstrating that the measured rotation directly equals intrinsic optical activity.

I also remain unconvinced by the way the manuscript positions ferroaxial approaches in comparison to the present mechanism. The authors repeatedly contrast their proposal with ferroaxial electric-field control and imply that the latter applies only to a relatively narrow class of materials. I do not think this comparison is sufficiently balanced. From a purely symmetry-based perspective, the difference in generality between the two approaches does not appear especially large: ferroaxiality is symmetry-allowed in 13 crystallographic point groups, whereas the linear piezochiral effect discussed here is allowed in 15 point groups. I therefore think the manuscript should moderate its comparative claims regarding the relative breadth or universality of the proposed approach.

Overall, I consider the symmetry concept and the underlying physical picture to be interesting and potentially important. However, for a journal such as Nature, where the expected downstream influence of the claims is exceptionally high, I believe that statements concerning the direct observation and quantitative extraction of strain-induced optical activity should be supported by a more rigorous propagation analysis. At present, I still see a gap between the demonstrated experimental phenomenology and the strength of the central optical interpretation.

Referee #3

(Remarks to the Author)
When I consider the discussion between Reviewer 1 and the authors, it appears to me that the concerns raised by the Reviewer in the previous round have been addressed satisfactorily by the authors. What now remains are previously unraised doubts regarding the unambiguous experimental identification of chirality. A central issue is that the chiral response is intermixed with linear birefringence, and chirality is never measured independently of this background contribution. For the reviewer, the ambiguity of the experimental analysis is further reinforced by the fact that birefringence and chirality together give rise to a complex propagation behavior. I agree with the described phenomenology.
The authors address this issue by conducting their analysis partly on a qualitative basis. The chirality manifests itself clearly through the investigated sign reversals and through their absence in non-chiral configurations. Certain effects of the birefringence average out in the analysis. For a qualitative demonstration of strain-induced chirality, I would regard this as sufficient, even though background-free chirality is not shown and the explicit quantitative propagation behavior resulting from the superposition of birefringence and chirality is not calculated. I would expect a fully quantitative treatment to appear in subsequent, more technically focused studies. This would also have to answer the question what chirality is worth when superimposed by a strong birefringent contribution.
In addition to the analysis discussed above, the authors also demonstrate other consequences of the proposed strain dependence, such as a quadratic dependence and a characteristic dispersion behavior. Overall, I thus consider the reported results to provide convincing evidence for piezochirality.
The subsequent comments by Reviewer 1 concerning the comparison between ferroaxiality and piezochirality appear, according to the Reviewer’s own wording, to be of secondary importance and mainly related to phrasing. A minor reformulation should be sufficient to resolve this issue.

Open Access This Peer Review File is licensed under a Creative Commons Attribution 4.0 International License, which permits use, sharing, adaptation, distribution and reproduction in any medium or format, as long as you give appropriate credit to the original author(s) and the source, provide a link to the Creative Commons license, and indicate if changes were made.
In cases where reviewers are anonymous, credit should be given to 'Anonymous Referee' and the source.
The images or other third party material in this Peer Review File are included in the article's Creative Commons license, unless indicated otherwise in a credit line to the material. If material is not included in the article's Creative Commons license and your intended use is not permitted by statutory regulation or exceeds the permitted use, you will need to obtain permission directly from the copyright holder.
To view a copy of this license, visit https://creativecommons.org/licenses/by/4.0/

Response to Referee #1

- The manuscript claims that mechanical strain can induce chirality in a solid that is otherwise achiral. This is an appealing idea, and strain engineering is indeed an important tuning approach in condensed matter physics. However, in its current form the paper does not yet meet the standards required for Nature. The evidence for a genuine chirality transition is not convincingly established, and the manuscript does not sufficiently address relevant existing literature where similar effects have already been investigated.

We appreciate the Referee's generally positive remark. We understand that they remain unconvinced about some aspects of our manuscript. Below we endeavor to address all concerns point by point. We expect that the Referee will be pleased with the revised version of the manuscript.

- The novelty of the work is not clearly supported by the literature discussion. There already exist studies where external fields drive a transition from an achiral state to a chiral one. In particular, electric field control of chirality in achiral ferroaxial NiTiO3 has been demonstrated. (Phys. Rev. Mater. 5, 124409 (2021)). Strain stabilized chiral phases have been reported in BaTiS3 (Adv. Funct. Mater. 35, 2416560 (2025)). Pressure and strain tunability of achiral to chiral transitions has been analyzed for K3NiO2 (Phys. Rev. B 111, 174102 (2025)). A systematic framework for such displacive chiral transitions has also been developed recently (arXiv 2503.13076v1). These works must be cited and the authors should clarify what new principle is established in the present study that goes beyond them.

We thank the Referee for his careful review of the literature. Indeed, some progress has been made recently in the external control of structural transitions between achiral and chiral states, and we have added these references to the list of citations in the revised manuscript. Yet, we think our work is distinct, expanding on what has been done before and most importantly placing piezochirality on a significantly more solid foundation.

1. Previous reports discussed the effect of external pressure or strain to amplify a chiral instability without selecting the handedness. Compared with these studies, the piezochiral effect discussed here enables deterministic selection of the handedness of the induced chirality, controlled by both the direction and the sign of the applied strain.
2. Electric field control of chirality is restricted to axially ordered systems, which constitute a relatively narrow class of materials. The piezochiral effect discussed here, which is based on unbalancing compensated enantiomers of opposite handedness, applies instead to a broad class of non-centrosymmetric materials.

Furthermore, in electrically conducting systems electric field control introduces significant dissipation, which is unfavorable for practical implementations. Strain can be applied with minimal dissipation in both insulating and conducting systems.

Taken together, the piezochiral effect provides a distinct and general framework for the control of chirality, enabling both deterministic and dissipation-free operation.

- An issue concerns the symmetry analysis. To demonstrate that chirality has emerged, it must be shown explicitly that all improper symmetry operations such as mirror and inversion are removed by the applied strain. The manuscript states that the symmetry changes to a chiral point group but does not specify which symmetry elements are broken in each strain configuration. Without a clear and complete account of the symmetry lowering process, the optical rotation data may reflect only strain induced anisotropy or local distortions rather than true crystallographic chirality.

We agree that a clear and explicit symmetry analysis is essential. Table R1 summarizes a complete analysis of the broken symmetry operations for each strain configuration, explicitly showing how the parent achiral point groups are reduced to chiral subgroups. This information has been added to the revised Supplementary Information.

In addition, we note that strain-induced anisotropy alone induces only birefringence but does not produce optical activity. Our experimental scheme takes care of this effect by measuring polarization rotation signals for a 360° sweep of the incident light polarization, where birefringence-induced rotations average out, leaving pure optical activity arising from chirality (see also the next point).

Parent Point Group	Applied Strain Components	Broken Symmetry Operations	Resulting Subgroup
$\bar{4}2m$ (D2d)	$\varepsilon_{xx}/\varepsilon_{yy}$	$\bar{4}^{+}, \bar{4}^{-}, m_{110}, m_{1\bar{1}0}$	222 (D2)
$mm2$ (C2v)	ε_{xy}	m_x, m_y	2 (C2)
$\bar{4}$ (S4)	$\varepsilon_{xx}/\varepsilon_{yy}/\varepsilon_{xy}$	$\bar{4}^{+}, \bar{4}^{-}$	2 (C2)
m (Cs)	$\varepsilon_{xy}/\varepsilon_{yz}$	m_y	1 (C1)

Table R1 | Strain-induced symmetry reduction. Applied strain components that eliminate all improper symmetry operations are listed for each parent achiral point group, along with the broken operations and the resulting chiral subgroup.

- Experimentally, there is no demonstration of reproducibility, no temperature or wavelength dependence, and no discussion of possible systematic effects like strain induced birefringence. For claims of this importance, validation through multiple independent measurements is needed.

  The statistical reporting is insufficient. Error bars are not defined in the figures, the number of repeated measurements is unclear, and no quantitative assessment of uncertainty or significance is presented. Given the small magnitude of the reported rotation, this is a substantial weakness.

This is a well taken point and we are happy to follow up on this criticism. In the revised manuscript, we provide a discussion and quantitative assessment of the statistical robustness of our measurements by the following analysis of the experimental uncertainty.

Fig. R1a shows the detected rotary power under uniaxial strain along the *x* direction. Error bars, indicating the standard error of the mean, are smaller than the data points. The corresponding relative uncertainty, normalized to the maximum signal amplitude (5.05 deg cm^{-1}), is plotted in Fig. R1b and found to be well below 1% of the largest observed rotary power.

Reproducibility is demonstrated in Fig. R1c, comparing data sets of two independent strain-scan measurement performed under the same conditions. The normalized difference between the two measurements is of the order of 1%, as shown in Fig. R1d. The same set of analyses for strain applied along the *y* direction is provided in Fig. R2.

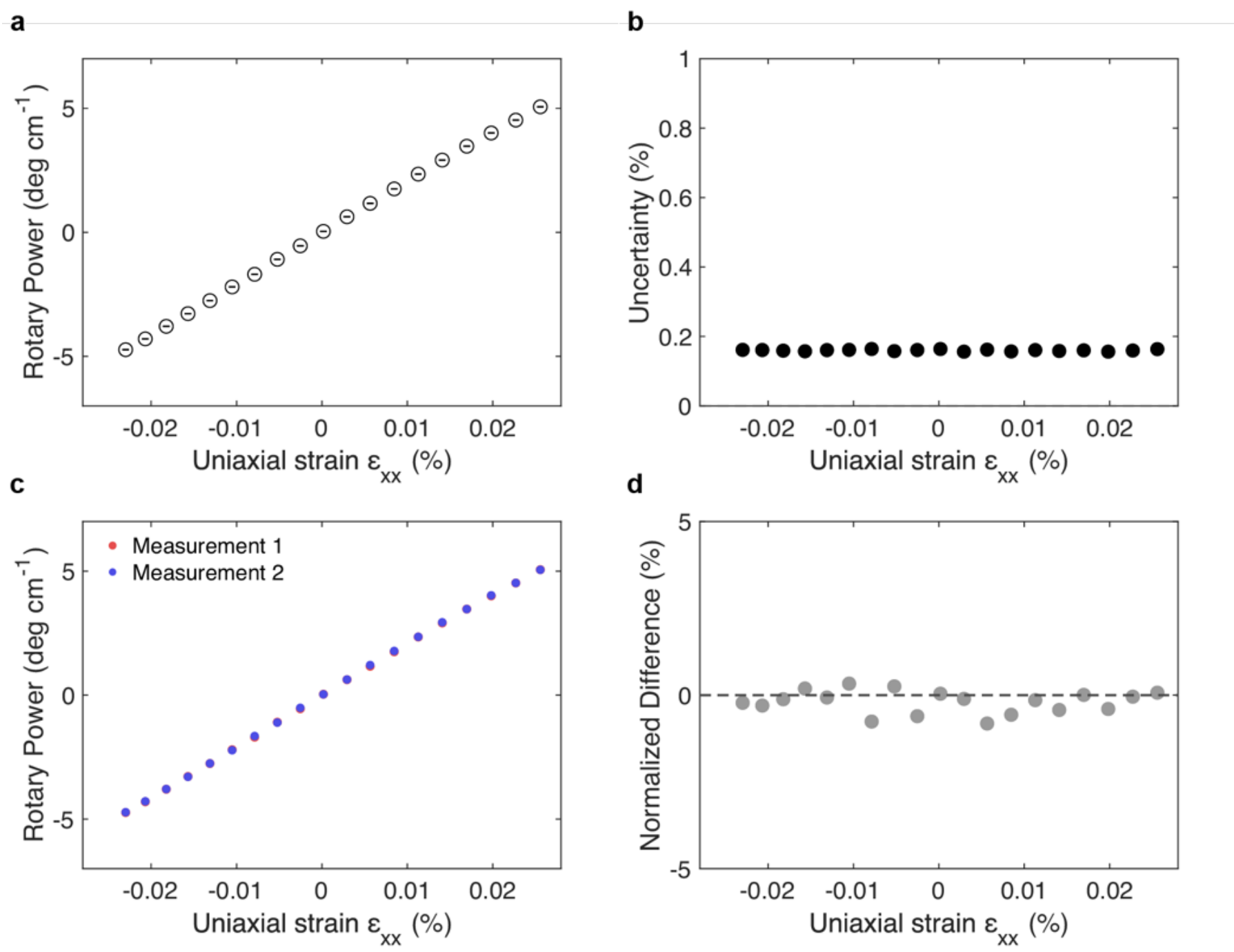

Fig. R1 | Rotary power under uniaxial strain along the *x* direction. a, Rotary power signal with error bars, defined as the standard error of the mean, indicating the experimental uncertainty. **b,** Measurement uncertainty corresponding to panel a, normalized by the maximum rotary power (5.05 deg cm^{-1}). **c,** Rotary power signal measured in two independent scans under the same strain conditions. **d,** Normalized difference between the two scans shown in panel c.

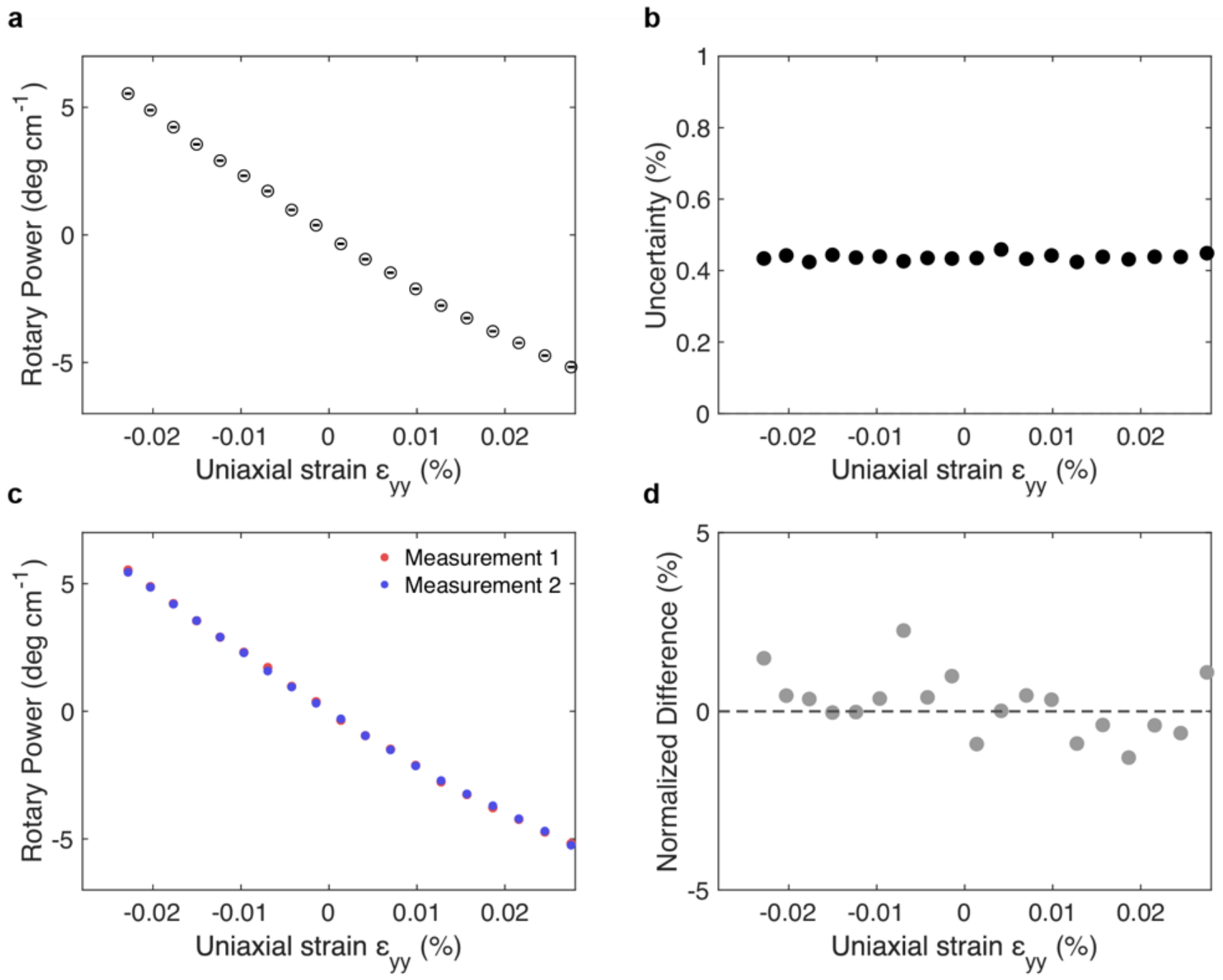

Fig. R2 | Rotary power under uniaxial strain along the *y* direction. a, Rotary power signal with error bars, defined as the standard error of the mean, indicating the experimental uncertainty. **b,** Measurement uncertainty corresponding to panel a, normalized by the maximum rotary power (5.05 deg cm^{-1}). **c,** Rotary power signal measured in two independent scans under the same strain conditions. **d,** Normalized difference between the two scans shown in panel c.

Potential systematic contributions, such as strain-induced birefringence, are carefully accounted for by measuring the polarization rotation signal for a 360° sweep of the incident probe light polarization and extracting the strain-induced optical activity by averaging the signal over all these angles.

In brief, strain-induced birefringence contributes a second-order effect to the polarization rotation signal. When a linearly polarized pulse traverses a birefringent crystal, the outgoing beam becomes elliptically polarized. The projection on the *x* and *y* axes (the balancing axes for the incoming polarization) will differ depending on the relative angle between the incoming polarization and the fast axis of the birefringent material. As illustrated in Fig. R3 below, the resulting signal exhibits a four-fold symmetry with respect to the incoming probe light polarization. In contrast, the contribution from optical activity is independent of the polarization of the incident probe. The total polarization rotation signal θ takes the form

$$\theta(\varphi) = \frac{\pi d}{\lambda_0 \sqrt{\varepsilon_{11}}} c - \frac{\pi^2 d^2}{2{\lambda_0}^2 \varepsilon_{11}} \sin(4\varphi) b^2 \,,$$

where φ is the incident polarization angle, d is the propagation distance, λ_0 is the probe wavelength, ε_{11} is the in-plane dielectric constant, c characterizes the optical activity, and b quantifies the birefringence. The pure optical activity can be extracted by averaging the signal over all the incoming polarization.

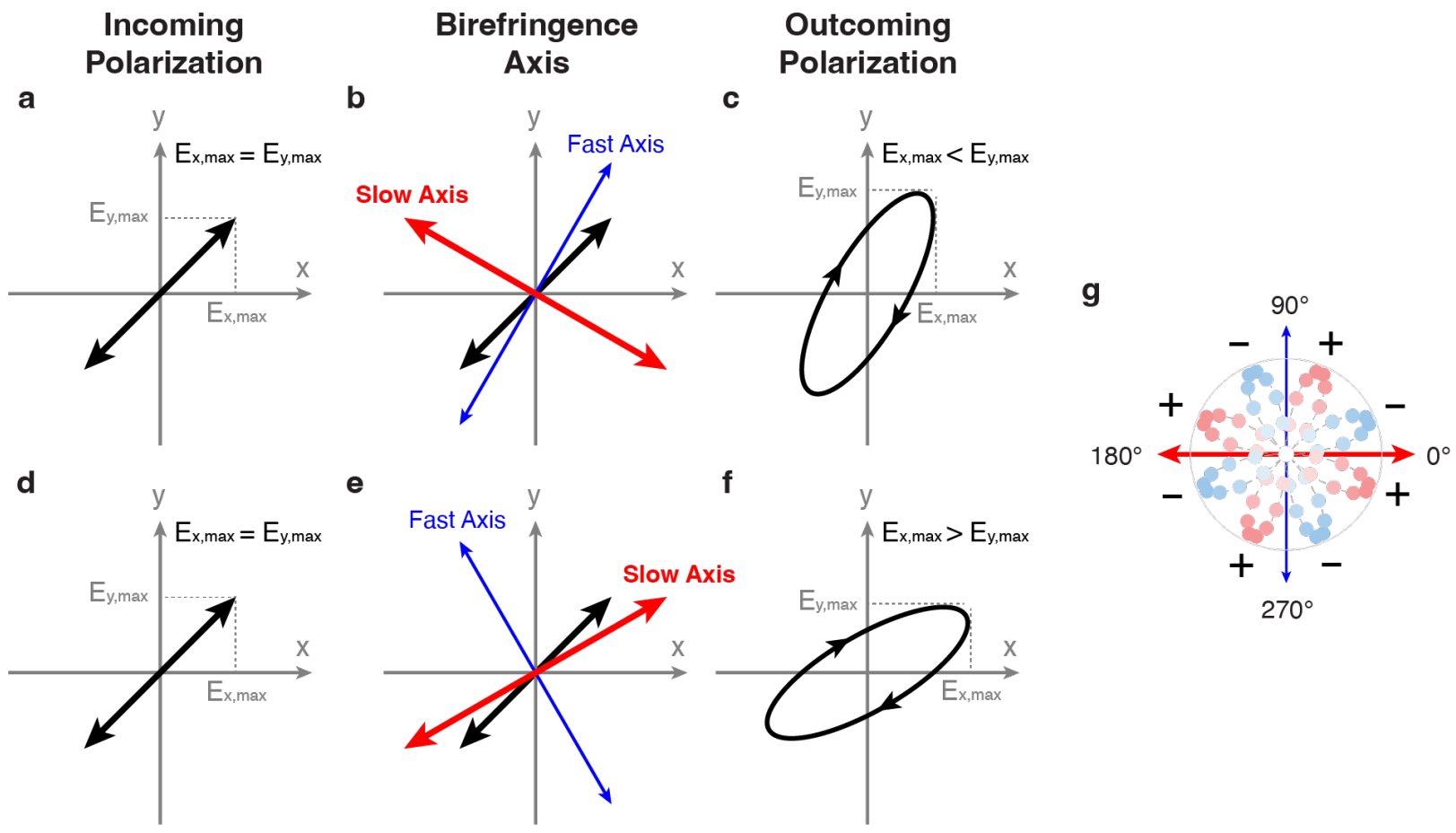

Fig. R3 | Illustration of the contribution of birefringence to the polarization rotation signal. a, Linearly polarized incoming polarization. X and Y axes are set to balance the signal on the two detectors. **b,** Orientation of the birefringence axis relative to the incoming polarization with fast axis closer to the incoming polarization. **c,** The outcoming polarization, where the projections on the two detectors differ. **d-f,** Orientation of the slow axis closer to the incoming polarization. The sign of the signal is reversed. **g,** Polarization rotation signal as a function of the incident polarization angle.

Moreover, we followed the Referee's suggestion and performed additional measurements using a second probe wavelength of 633 nm. The experimental results are summarized in Fig. R4. We observe strain-induced optical activity with the same qualitative behavior as observed for the 808 nm probe, confirming the robustness of the effect.

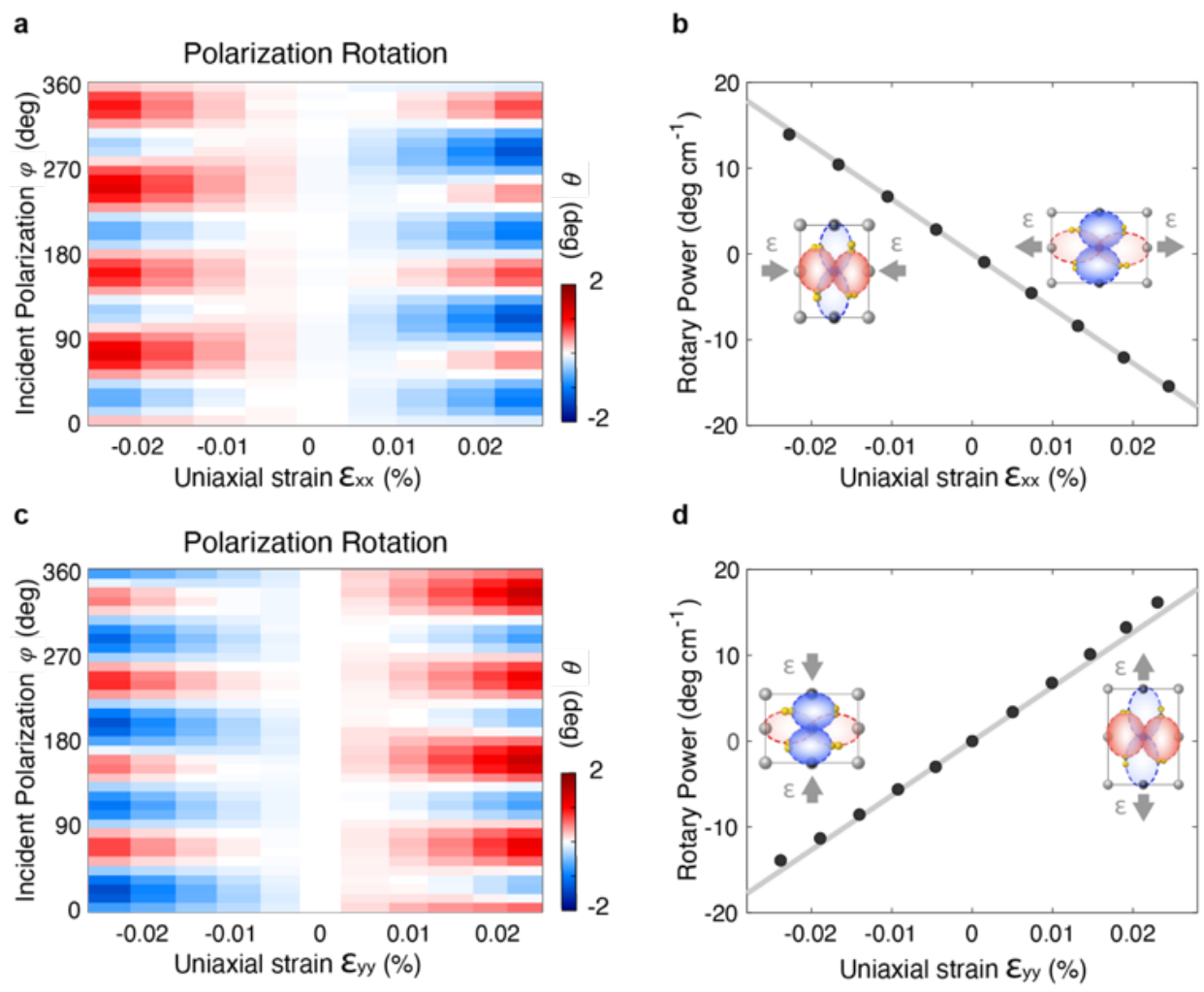

Fig. R4 | Polarization rotation measurements with 633 nm probe. a, Polarization rotation signal for uniaxial strain along the x direction for different polarization angles of the incident probe. **b,** Rotary power as a function of uniaxial strain, extracted from (a). **c,** Same as (a), but for uniaxial strain along the y direction. **d,** Same as (b), but for uniaxial strain along the y direction.

As shown in Fig. R5, the rotary power measured at 633 nm is approximately 3 times larger than that found at 808 nm. This enhancement is consistent with the general behavior of optical activity in chiral materials, which increases as the probe wavelength approaches the electronic resonance. In $AgGaS_2$, the band-gap energy is 2.76 eV, corresponding to a wavelength of approximately 450 nm[1].

More quantitatively, the rotary power in a chiral crystal is generally described by the Lorentz optical rotatory dispersion expression[2]

$$\alpha(\lambda) = \frac{\pi C_j \lambda}{2n}\left(\frac{1}{\lambda^2 - \left(\lambda_j^L\right)^2} - \frac{1}{\lambda^2 - \left(\lambda_j^R\right)^2}\right),$$

where j is the index for the different electronic resonances, C_j are material constants, n is the refractive index, λ_j^L and λ_j^R are resonance wavelengths for left- and right-circularly polarized light.

For an estimate, we consider the band-edge interband transition, which is the nearest resonance to the probe wavelengths, hence dominates the optical rotatory dispersion. Assuming a small circular splitting, $\lambda_{L,R} = \lambda_0 \mp \delta$ ($\delta \ll \lambda - \lambda_0$). In this case,

$$\alpha(\lambda) \propto \frac{\lambda}{(\lambda^2 - \lambda_0^2)^2}$$

Taking parameters for $AgGaS_2$ with $\lambda_0 = 450\ nm$, this single-resonance estimate yields

$$\frac{\alpha(633\ nm)}{\alpha(808\ nm)} \approx 4$$

In real crystals, additional interband transitions and finite linewidth broadening will reduce the enhancement predicted by the simplified single-resonance model, bringing it into agreement with the experimentally observed threefold increase.

These additional measurements and the corresponding analysis have been included in the revised Supplementary Information.

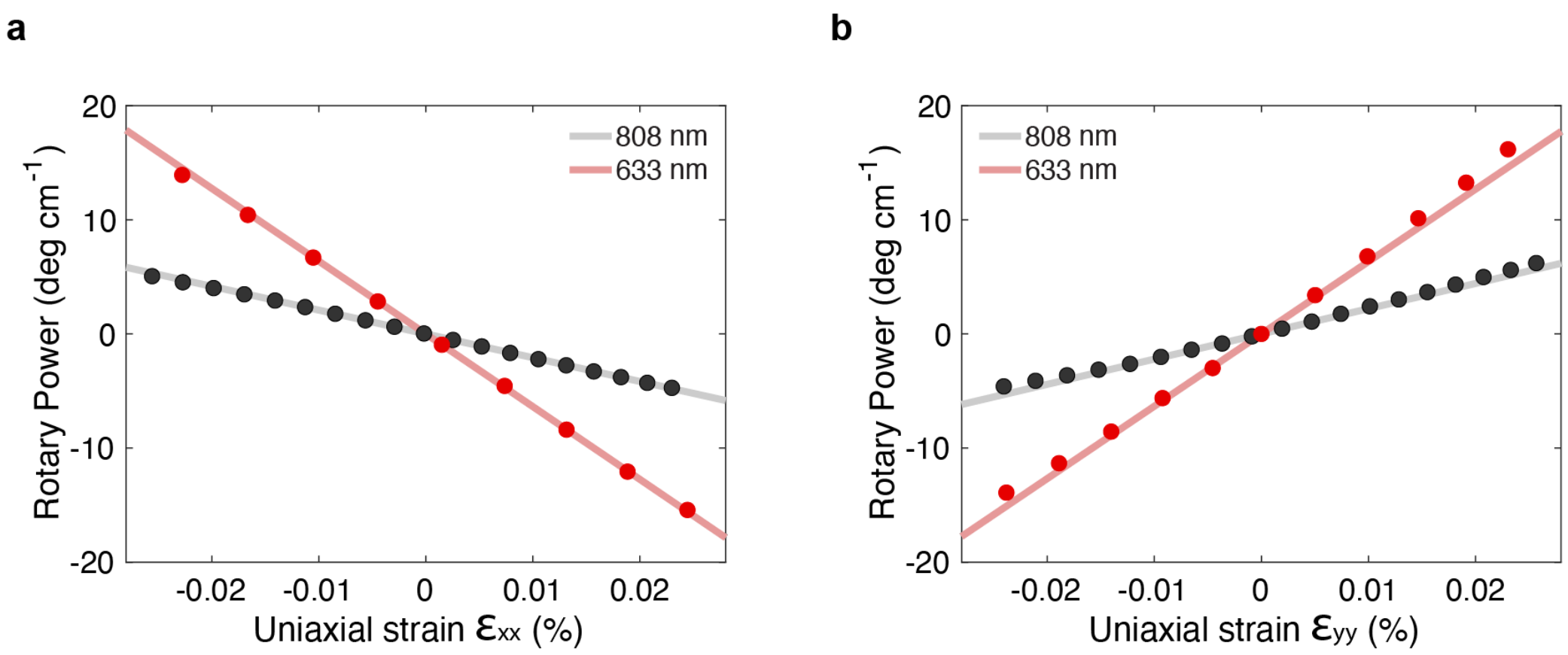

Fig. R5 | Comparison between measurements of two wavelengths. a, Rotary power as a function of uniaxial strain along crystal *a* axis for two wavelengths. **b,** Rotary power as a function of uniaxial strain along crystal *b* axis for two wavelengths.

To further examine possible systematic uncertainty in the measurement, we performed identical strain-dependent polarization rotation measurements on a reference sample that is not expected to be peizochiral.

We selected CaF_2, with point group $m\bar{3}m$ (Oh). Due to the presence of inversion symmetry, the piezochiral effect is symmetry-forbidden. The CaF_2 crystal with a (001) surface was prepared with the same thickness as the one from $AgGaS_2$ samples. We applied uniaxial strain along [010] direction, and the measurements were conducted under identical optical conditions using the 808 nm probe beam. Three independent measurements were conducted at different positions on the CaF_2 sample surface (Fig. R6). The extracted strain-induced rotary power was one order of magnitude smaller compared to that measured in $AgGaS_2$. At one sample position, we even found opposite slope of the strain amplitude dependent rotary power.

We attribute these small and position-dependent signals to systematic experimental uncertainties, potentially arising from slight optical misalignment, minor strain inhomogeneity, or imperfections in the detection optics.

Given that the rotary power observed in $AgGaS_2$ exceeds this background by nearly an order of magnitude and shows a consistent sign and magnitude, these results support the interpretation that the strain-induced optical rotation in $AgGaS_2$ arises from an intrinsic piezochiral response of the material. We added this information to the revised Supplementary Information.

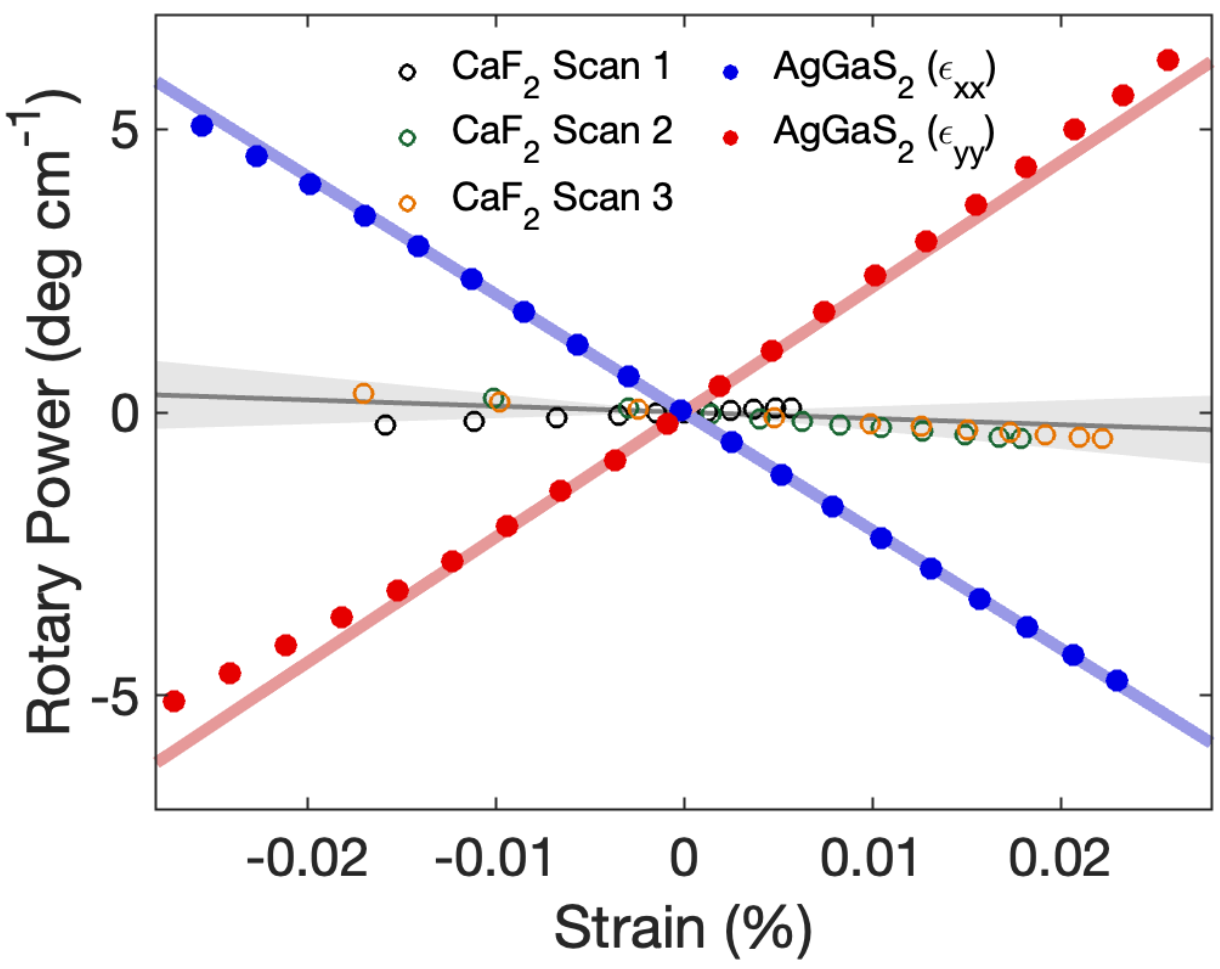

Figure R6 | Comparison between strain-induced rotary power between piezochiral $AgGaS_2$ and reference sample CaF_2. The shaded area represents the standard deviation of the fits from three measurements. The black line represents the average of linear fits obtained from the three CaF_2 measurements, while the shaded region denotes the corresponding standard deviation.

Lastly, we believe that a temperature dependent measurement is not very important. In fact, $AgGaS_2$ does not undergo any structural or electronic phase transition in the experimentally accessible temperature range, and the piezochiral response in $AgGaS_2$ sample is not expected to exhibit a significant temperature dependence.

- In summary, the direction is interesting and potentially important, but major revisions are needed before this manuscript could be considered for publication in Nature. Explicit differentiation from existing studies including those cited above, more rigorous symmetry analysis, and significantly strengthened experimental evidence would be required to support the central claims.

We thank the Referee again for their constructive review. In the revised manuscript, we have expanded our discussion of previous relevant studies and highlighted the novelty of our work. We have also strengthened our symmetry analysis, both in the main text and in the Supplementary Information. On the experimental side, we now describe in detail how our comprehensive measurement protocol isolates the pure optical activity, ruling out birefringence contributions arising from anisotropic strain. We hope that these amendments address all the Referee's concerns and are hopeful of a positive recommendation.

Response to Referee #2

- In this article, the authors develop on, and prove the existence of a new crystal property coined piezochiral effect, which refers to the induction and control of chirality in achiral crystalline materials through strain (similarly to piezoelectricity for polarization).

  After a definition of the piezochiral tensor, they first analyse which crystal point groups can host this effect through a formal symmetry and group theory approach. The authors demonstrate that uniaxial strain can induce chirality in materials with specific crystallographic point groups that they list. From this list, they could identify the model system AgGaS2 (point group -42m) where they show the existence of this piezochiral response through both experiments and DFT calculations. Both clearly show the presence of a linear evolution of chirality amplitude versus uniaxial strain amplitude in AgGaS2, which means that chirality change sign with the sign of the strain (i.e. compressive or tensile). From the simulation side, by defining a chiral order parameter (a pseudoscalar function depending on atomic positions that reflects handedness) to quantify chirality and check how it evolves versus uniaxial strain in AgGaS2. From the experimental side, they do so by measuring the rotatory power of a linearly polarized light through the sample with respect to uniaxial strain.

  As both, theory and experiment, confirm the same linear evolution of the induced chirality versus uniaxial strain in AgGaS2, it gives a very strong confirmation and validation of the existence of the piezochiral effect. The authors also discuss higher-order piezochiral couplings and the integration of piezochiral materials into microelectromechanical systems (MEMS) for on-chip chirality manipulation.

  The discovery clearly opens new avenues for chirality control in solid-state systems, with potential applications in spintronics, asymmetric catalysis, biosensing, or quantum materials.

- The paper is clearly written, it is sufficiently well popularized for a boarder audience of non-specialists, it introduces a new concept never observed or proved before, it opens new avenues as this is quite novel with broad interdisciplinary implications, and it combines carefully done symmetry analysis, first-principles simulations, and experimental validation such that I think it checks all the points to be published in Nature journal. I have the following comments that should be responded by the authors before acceptance, but I think they are quite easy to respond.

We thank the Referee for this positive assessment of our work. We appreciate their recognition of the novelty, clarity and broad impact of the piezochiral effect, and the support for publication. Below, we will address the specific comments raised.

- 1) The authors demonstrate the presence of a piezochiral response in achiral space groups. However, why not in chiral space groups? Intuitively, one would expect a piezochiral response in chiral space groups as well, analogous to how piezoelectricity exists in polar systems, or did I missed something on the way of why a focus is done on achiral systems? Addressing this could further generalize the scope and impact of the paper.

Indeed, the piezochiral effect is not restricted to achiral space groups. According to the symmetry-based definition introduced in our work, the piezochiral coupling tensor is also allowed in all chiral space groups.

In the present manuscript, however, we focus on achiral systems, because the handedness of the strained state in those materials can be controlled by the direction and sign of the applied strain. This tunability makes achiral systems particularly attractive for demonstrating strain-controlled switching between left and right handedness and for realizing reversible chiral functionalities. In contrast, starting from an equilibrium chiral state would primarily allow to modulate the chiral order parameter, which we believe is less attractive for potential applications. Nevertheless, this would be a straightforward extension of what discussed here and we expect that future studies by our or other groups will tackle selected examples.

We followed the Referee's suggestion and have clarified this point in the revised manuscript. In addition, we have included a systematic classification of the piezochiral coupling for all the crystallographic point groups in the Supplementary Information.

- 2) It is, in a way, related to my comment 1, but I think there are already some previous works playing with strain to tune chirality in chiral crystals, for example in PRB 102, 235127 (2020) or with pressure (which is in the way the converse effect of strain) in J. Phys. Soc. Jpn. 43, pp. 195-203 (1977). I think the authors should extend their introduction regarding previous works that played with strain or stress and chirality or related properties, even if this was not called "piezochirality" it is nevertheless very close/related to the present work.

We thank the Referee for pointing out these earlier works on the interplay between strain, pressure and chirality. We have now included these references into the revised manuscript, at the same time highlighting the benefits of our work.

- 3) For the symmetry analysis, I would have liked to also see what are the symmetry that are broken in each point groups and what are the associated subgroup, could the authors add such information if they also think it can be useful (probably in SI as it would be of interest to more specialist than board audience?)?

We followed the Referee's suggestion. As also requested by Referee #1, we have now included this information in the revised Supplementary Information.

- 4) Upon carefully reviewing the literature to determine whether the term piezochirality is indeed novel, we, however, can find such a mention in J. Phys.: Condens. Matter 37, 163004 (2025). In that article, the term "piezochirality" was anticipated as a potential crystal response, but no experimental nor computational nor symmetry evidence was provided to confirm its existence as done here. This prior mention does not reduce the novelty and impact of the present study, which rigorously establishes the effect through symmetry analysis, DFT calculations, and experimental validation. However, for completeness and fairness, I think it would be appropriate to acknowledge this anticipation in the discussion.

We take this point and have cited this paper.

- 5) I found this typo in the bibliography: Refence 19, "mangamse" should be "manganese"

We thank the Referee for the careful reading and have corrected this typo.

- 6) Why is there two references lists in the SI with the same numbering? This is confusing, the authors should clean it.

This was done by mistake, and the issue has been resolved.

- So, beside a careful check of previous literature (i.e. put in better perspective previous works w.r.t. the present work) as a major comment, I think the paper fit very well with Nature publication rules.

Again, we thank the Referee for the positive recommendation.

Response to Referee #3

- The authors of this work show how the application of strain deletes all the mirror planes of a material, hence introducing a chiral state. Its sign can be selected through the sign or the direction of the strain, and its presence is demonstrated in strain-dependent measurements of the optical activity. The relation between strain and chirality is denominated as piezochiral effect.
- The key point of this manuscript is to show that chirality in a material can be generated and switched in a controlled manner. This is a fundamental and straightforward idea, and apart from the points listed below, its derivation and demonstration are very convincing. Likewise, introduction of the piezochiral effect is fundamental and clear. Even if I have some concerns about novelty and generality, I would support publication if the following points can be mended.

We thank the Referee for the positive remarks on the significance of our work and the support towards publication. We address the points raised below.

- (1) There is a larger context than the one given by the authors. There are quite a number of examples in the literature where the controlled creation of chiral states has been discussed. The authors mention Refs. 11-13. Other examples where the topic is covered (there are more!) are found in Phys. Rev. B 111, 174102 (2025) (K3NiO2 and related oxides; see Refs 28, 29 in this one), Chem. Eur. J. 23, 7990 (2017) (metal-organic frameworks), Science 384, 1096 (2024) (nematic systems), Microscopy and Microanalysis 30, 1409 (2024) (quasi-1D BaTiS3). A review in Materials 15, 5812 (2022) discusses how continuous structural parameters (e.g., internal coordinate u) can be tuned, including by external pressure, to move smoothly between achiral and chiral configurations. I agree that all these are different from the present work, and the piezochiral behavior is not discussed. Nevertheless the authors should put their contribution in relation to this considerable body of work.

We appreciate the Referee's careful review of the literature on chirality control in various materials. In the revised manuscript, we have cited the suggested papers to provide a more comprehensive overview of earlier efforts. We have also clarified the significance of our work within this context.

- (2) I cannot verify Eq. (2) as the specific form of Eq. (1) for -42m symmetry. In that point-group symmetry, group-theoratical tables show that we have T_yy = Txx and epsilon_yy = -epsilon_xx. Therefore, C = T_xx epsilon_xx + T_yy epsilon_yy = 0. In particular, the distinction between epsilon_xx and epsilon_yy made in Eq. (2) does not make sense to me because of the negative equality of the two.

We believe the Referee is not right here, as we clarify below.

Based on Neumann's principle, the piezochiral coupling

$$C = \sum_{ij} T_{ij} \varepsilon_{ij} \text{ (1)}$$

must remain invariant under all symmetry operations of the parent point group.

In systems with $\bar{4}2m$ symmetry, we consider chirality induced by uniaxial strain with components ε_{xx} and ε_{yy}, which gives

$$C = T_{xx}\varepsilon_{xx} + T_{yy}\varepsilon_{yy} \text{ (2)}$$

Applying the four-fold roto-inversion operation ($\bar{4}$) to both sides of the equation results in a sign reversal of the chirality on the lefthand side, while ε_{xx} and ε_{yy} are interchanged on the righthand side. This yields

$$-C = T_{xx}\varepsilon_{yy} + T_{yy}\varepsilon_{xx}. \text{ (3)}$$

A comparison of Eqs. (2) and (3) shows that consistency is fulfilled only when $T_{xx} = -T_{yy}$, showing that the two tensor components are opposite in sign.

This analysis does not follow standard group-theoretical tables because the piezochiral tensor is not a conventional 2nd rank symmetric tensor. Instead, it is a 2nd rank symmetric pseudo-tensor, which is invariant under time reversal, but changes sign under inversion operation.

In response to the Referee's concern, we have included a systematic table of all the allowed piezochiral tensor components for every crystallographic point group in the revised Supplementary Information.

- (3) Little is said about the DFT calculations. For a verification of the chiral degeneracy, it is not needed: here the discussion of symmetry is sufficient. DFT produces the order of magnitude of the effect, but aside from a reference to Fig. 3 it is not discussed.

We have expanded the discussion of the DFT workflow in the revised manuscript.

- (4) The term "incident polarization" is repeatedly used. Polarization is not incident, however -- light is.

We have corrected it throughout the revised manuscript to "polarization of the incident light".

- (5) I find Fig. 2 confusing. I do not see how the atoms in panels a, b, c correspond to one another, and I do not see the antichiral relation between the upper and the lower half of the unit cell in panel a. What exactly do the lobes in panel c represent (what are the dashed lines?), and how does this correspond to panels a and b?

We thank the Referee for raising this point, which obviously requires clarification. We revised Figure 2 accordingly, which we also show below for the Referee's convenience. Panels **a** and **b** show the lattice structure of $AgGaS_2$ from two complementary viewv, one in perspective and one along the crystal c axis. The four lobes in panel **b** illustrate the same four spiral substructures of different handedness already shown in panel **a**.

Panel **c** provides the definition of the order parameter used to compute the values shown in panel **b**. It is defined as the sum of the triple products formed by all the normalized vectors connecting four neighboring atoms in a spiral structure. Since the definition of this order parameter is general and can be applied to other crystal systems, the atoms in panel **c** are not color-coded.

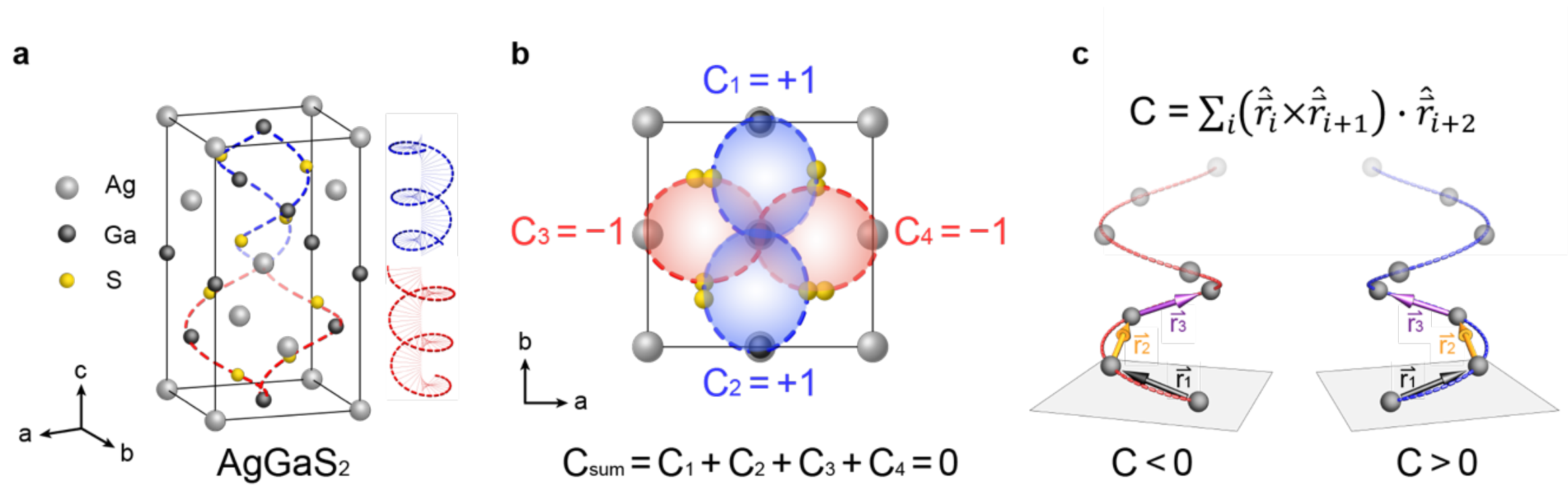

Fig. 2 | Static chiral order parameter in $AgGaS_2$. a, Unit cell of the antiferrochiral crystal $AgGaS_2$, composed of chiral substructures with opposite handedness. **b,** Normalized chiral order parameter of $AgGaS_2$. Substructures of opposite handedness yield opposite values, resulting in a vanishing net chiral order parameter. **c,** Definition of the chiral order parameter as the sum of triple products formed from normalized vectors connecting neighboring atoms along the spiral.

- (5) To me the conclusions appear as exaggerated. The effect demonstrated here is fundamentally important, but the effect is still rather special, and I do not see that broad spectrum of applications up to MEMS and on-chip applications. How would strain control be exerted on a chip? Also, the magnitude of the reported effect is quite small. For the applications discussed in lines 127-137 I do not see the need for strain-controlled chirality. To my impression, an intrinsically chiral constituent may be used just as well.

Whilst we think that the effect discussed here may become widely applicable, there is no point in overstating our case. We have adjusted the wording of the conclusions for improved clarity and outline our reasoning below in support of a broad outlook. We would like nevertheless to argue our case, and to push back some on the Referee's skepticism.

First, our symmetry analysis shows that piezochiral coupling is allowed in a wide class of materials, not only in the prototype compound studied here, but also in many systems that are routinely employed in MEMS platforms.

For example, GaAs, a standard material in MEMS technology, belongs to the point group -43m that allows higher-order piezochiral coupling.

Second, we would like to point out that on-chip strain generation and control is a mature technology. Precise and reversible strain engineering is available in MEMS platforms based on well-established actuation schemes, including electrostatic comb drives, thermomechanical actuators, and piezoelectric thin-film actuators. Thus, from an engineering standpoint, the integration of strain-controlled functionalities, including piezochirality, is realistic within existing platforms.

Third, regarding the magnitude of the effect, we note that the strain-induced optical activity observed in $AgGaS_2$ is less than one order of magnitude smaller than that of α-quartz, a widely used chiral optical material, for a modest strain of only 0.02%. Many crystalline systems that support piezochiral coupling, such as KH_2PO_4, can sustain strains values at or exceeding 1%, and therefore can be expected to show a substantially larger piezochiral response than in our case. Our present measurements should be viewed as a proof of principle rather than a reflection of the maximum attainable response across material classes.

Finally, with respect to applications such as asymmetric catalysis, we agree that conventional approaches rely on intrinsically chiral catalysts. However, a strain-switchable chiral environment may offer a novel and reversible control of enantioselectivity without replacing the catalyst. Such a capability could be advantageous in scenarios that require rapid alternation between enantiomeric outcomes or dynamic modulation of chirality under operating conditions. We therefore consider it appropriate to mention this potential direction enabled by the piezochiral effect introduced in this work.

We hope this response clarifies the rationale for including a discussion of the potential impact of our work. We also hope the Referee finds the revised presentation satisfactory.

References

1. Catella, G. C. & Burlage, D. Crystal growth and optical properties of AgGaS2 and AgGaSe2. *MRS Bulletin* **23**, 28-36 (1998).
2. Barron, L. D. *Molecular light scattering and optical activity*. (Cambridge University Press, 2009).

Response to Referee #1

- The manuscript proposes that mechanical strain can induce and control chirality in crystalline solids and introduces the concept of a piezochiral effect. The idea is appealing, and the combination of symmetry analysis, first-principles calculations, and optical measurements is potentially suitable for a broad audience. However, despite the authors' revisions and detailed response, I remain unconvinced that the central claims are established with the level of rigor required for publication in Nature.

We thank the Referee for recognizing the significance of our work. However, we respectfully disagree with the Referee's assessment that our claim falls short of the required level of rigor. On the theoretical side, the analysis is well established and provides clear predictions. Experimentally, these predictions are validated in the proposed material through strain-dependent measurements of optical activity. In addition, control measurements on a reference example show signals within the level of systematic uncertainty, in accordance with theoretical predictions. These results together provide a consistent picture supporting our central claim, and we are not sure what is needed in addition.

- My main concern relates to the identification of optical activity in the experiment. The authors interpret a polarization-independent component of the measured rotation signal as evidence for strain-induced chirality, arguing that birefringence contributions can be removed by averaging over the incident polarization angle. While this argument is formally valid under ideal conditions, it is not sufficient in practice. A polarization-independent offset is not uniquely indicative of optical activity and may arise from residual misalignment, imperfections in the polarization optics, or other systematic effects. The reference measurements on a centrosymmetric material show finite strain-dependent rotation signals, even though the effect should be forbidden by symmetry. The presence of such background signals raises concerns about whether the observed response in the target material can be unambiguously attributed to intrinsic optical activity. Moreover, no direct measurement of circular birefringence or related quantities is provided, so the identification of chirality remains indirect.

Indeed, it is important to distinguish intrinsic chirality induced effects from potential experimental artifacts. We are confident that the observed signal in $AgGaS_2$ originates from intrinsic piezochirality, based on the following experimental evidence.

- A polarization rotation signal clearly and reproducibly emerges when uniaxial strain is applied to $AgGaS_2$, a piezochiral compound by symmetry. The observed linear dependence on the applied strain is fully

consistent with the expected lowest-order linear piezochiral coupling in this material. Moreover, the signal reverses sign when strain is inverted between compressive and tensile.

- The sign of the polarization rotation signal also reverses when strain is applied along two orthogonal directions, in agreement with the theoretical expectation. We emphasize that great care was taken in the sample preparation and two stripe-shaped pieces with orthogonal in-plane directions were prepared from the same single crystal (see Fig. R1), ensuring identical thickness and out-of-plane orientation. The polarization rotation measurements were performed under identical experimental conditions, with only the strain direction and amplitude varied. While small systematic offsets may arise from imperfections in the experimental setup, the observed sign reversal, and its consistency under repeated measurements cannot arise from artefacts. This fact provides unambiguous evidence that the signal is intrinsic and originates from strain-induced chirality.

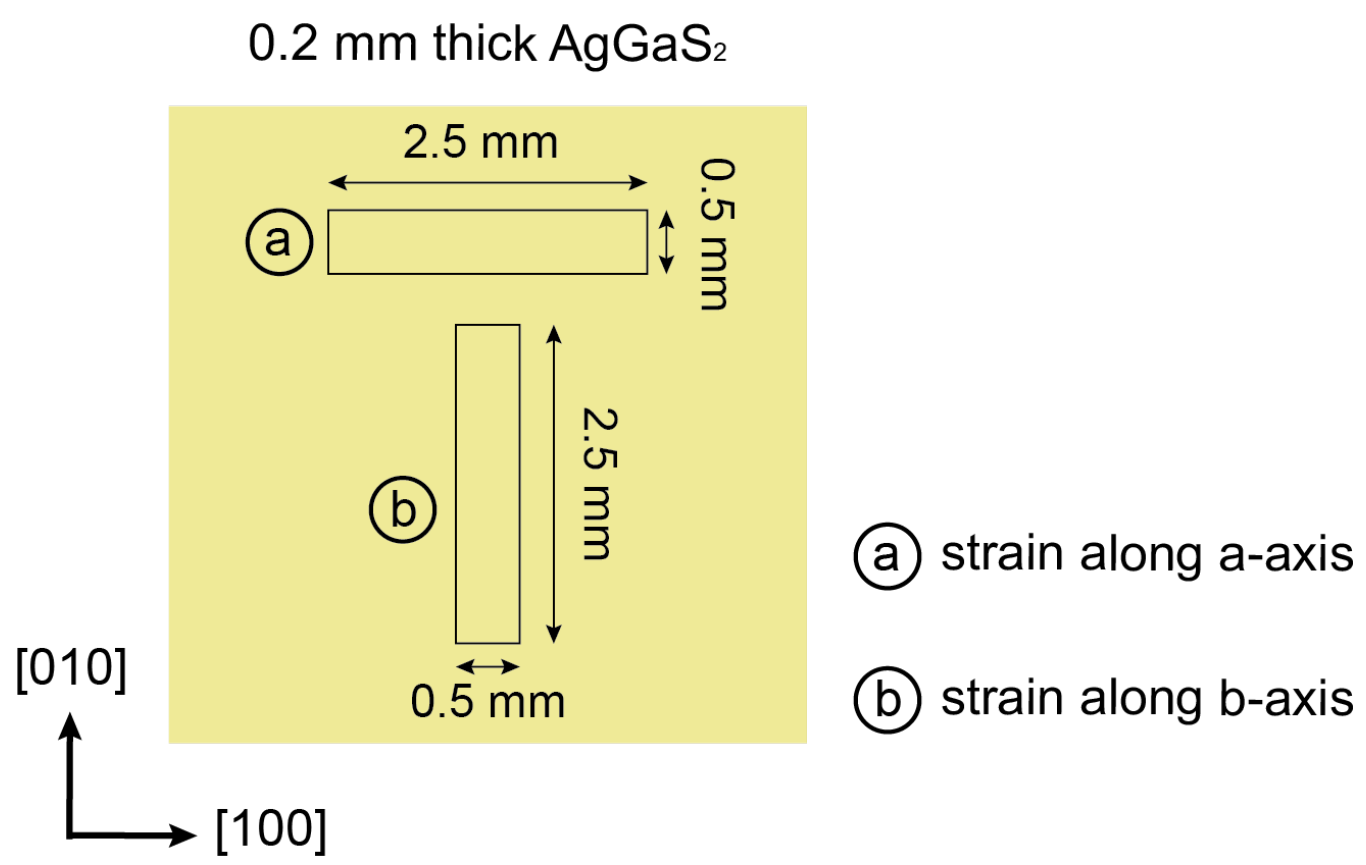

Fig. R1 | Schematic of sample preparation. Stripe-shaped pieces with their long axes along orthogonal directions were cut from the same single crystal.

- Following the Referee's earlier suggestion, we performed additional measurements using a different probe wavelength of 633 nm. The results exhibit the same qualitative behavior as observed for the originally used 808 nm probe, most notably a consistent sign reversal for orthogonal strain directions. The reproducibility of the effect at different probe wavelengths, together with the scaling of the detected amplitude in agreement with the theoretical expectations, further supports the robustness and intrinsic origin of the effect.
- As an additional check, we compared the strain-induced birefringence measured at two probe wavelengths to explicitly exclude potential birefringence-related artifacts. As shown in Fig. R2, the detected effective birefringence exhibits the same quadratic dependence on strain for both wavelengths, with a negligible difference in amplitude. The small difference is consistent with the known dispersion of the refractive indices for ordinary and extraordinary polarizations in $AgGaS_2$ (Fig. R3; *Applied Optics* **38**, 4577 (1999)).

Consequently, any artifact arising from birefringence at these two wavelengths would be expected to have a similar magnitude. This cannot account for the much larger difference observed in the measured optical activity, which clearly rules out birefringence as the dominant origin of the signal (Fig. R4). For clarity, the fitted coefficients for both the strain-induced birefringence and the optical activity are shown together in Table R1.

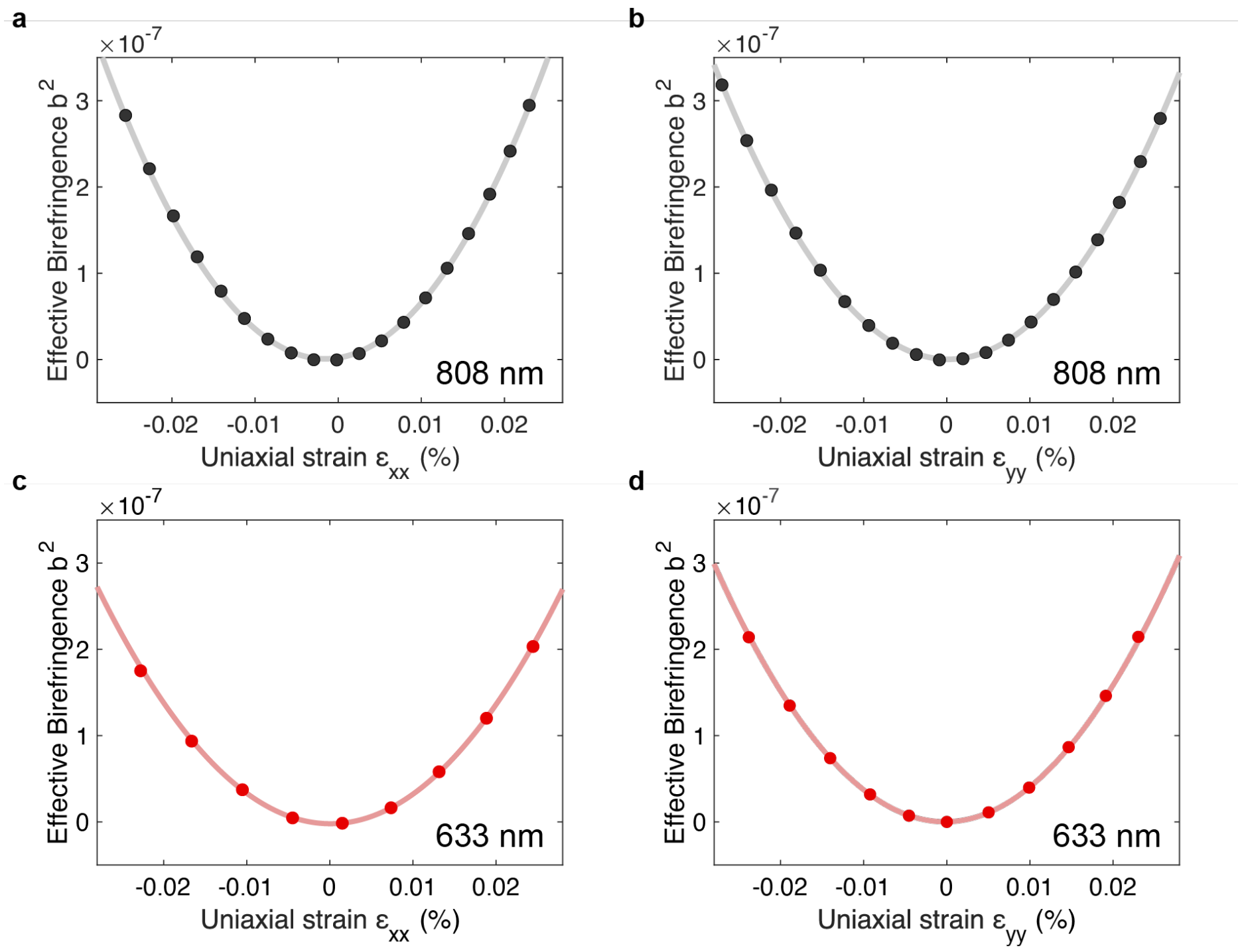

Fig. R2 | Comparison of measured strain-induced birefringence at two probe wavelengths. a, Square of the effective birefringence coefficient b^2 as a function of uniaxial strain along the crystal *a* axis, measured at 808 nm. **b,** Same as panel (a) for strain applied along the crystal *b* axis. **c,** Same as panel (a), measured at 633 nm. **d,** Same as panel (c) for strain applied along the crystal *b* axis. The data are fitted with a quadratic function $b^2 = \alpha(\varepsilon - \beta)^2$, where α is the coupling coefficient and β is the strain offset.

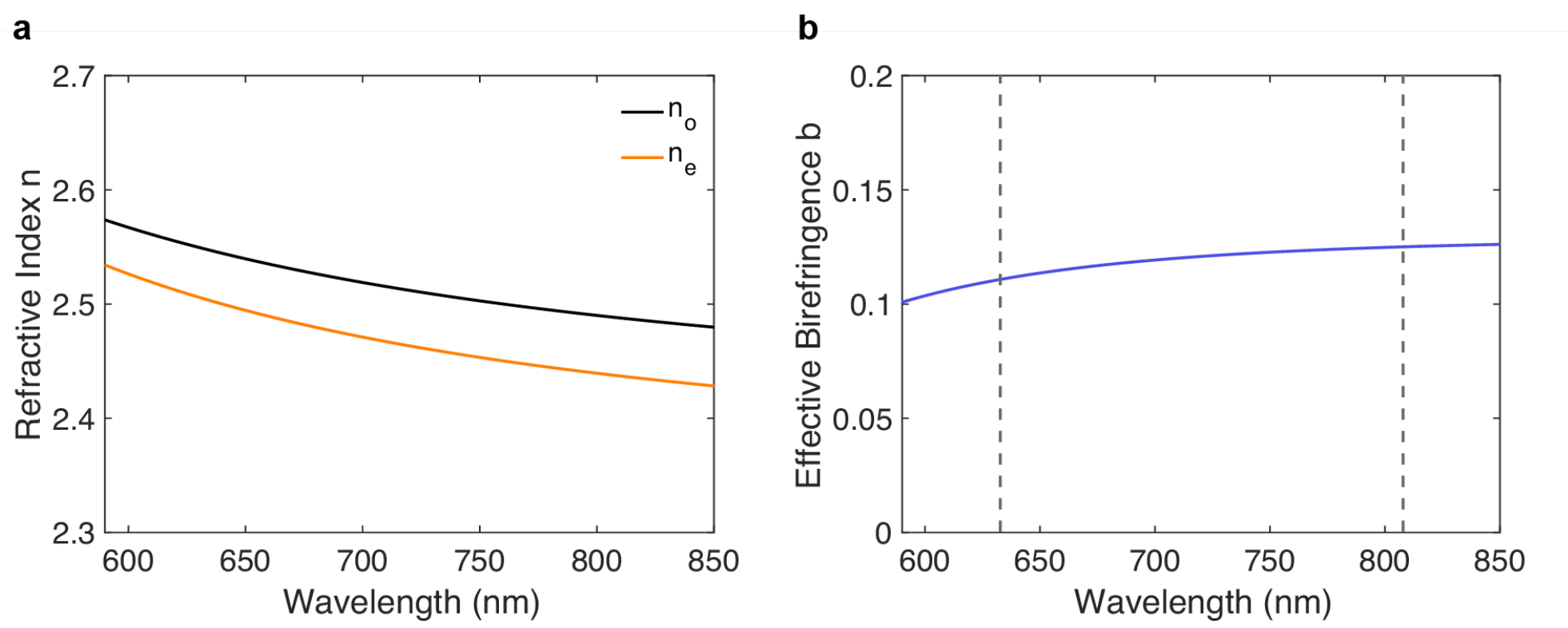

Fig. R3 | Wavelength-dependent birefringence in static $AgGaS_2$. a, Refractive index for ordinary (o) and extraordinary (e) polarizations, taken from *Applied Optics* **38**, 4577 (1999). **b,** Corresponding birefringence between the two polarizations. The dashed lines indicate the probe wavelengths used in the experiment.

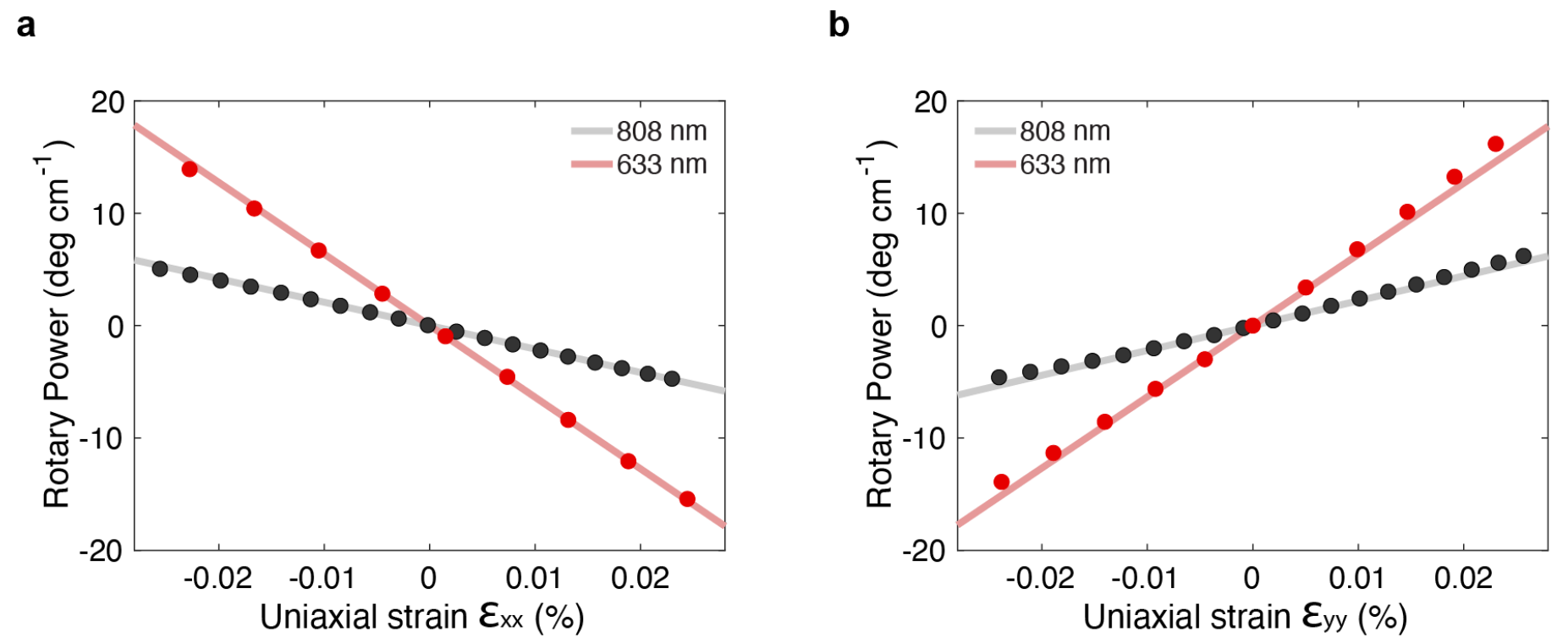

Fig. R4 | Comparison of strain-induced optical activity at two probe wavelengths. a, Rotary power as a function of uniaxial strain along the crystal *a* axis for two probe wavelengths. **b,** Rotary power as a function of uniaxial strain along the crystal *b* axis for two probe wavelengths. The data are fitted with a linear function $\rho = \gamma\varepsilon$.

Probe wavelength	Strain direction	Strain-induced birefringence α (1/unit strain)	Strain-induced optical activity γ (deg cm^{-1}/unit strain)
808 nm	ε_{xx}	1.36×10^3	-2.1×10^4
808 nm	ε_{yy}	1.39×10^3	2.2×10^4
633 nm	ε_{xx}	1.51×10^3	-6.4×10^4
633 nm	ε_{yy}	1.68×10^3	6.3×10^4

Table R1 | Coupling coefficients for strain-induced birefringence and optical activity. The parameters α and γ are obtained from quadratic and linear fits, respectively.

- Measurements on a reference sample, for which no piezochiral effect is expected from theory, are used to estimate the magnitude of potential systematic errors. The results are (1) significantly (at least one order of magnitude) smaller than the optical activity detected in the piezochiral material, and (2) show a position-dependent sign. In contrast, $AgGaS_2$ exhibits both robust signal magnitude and consistent sign under repeated measurements, even at different sample positions. This distinction further underscores how the residual signal in the reference sample arises from extrinsic effects such as inhomogeneous strain, whereas the response in $AgGaS_2$ reflects an intrinsic piezochiral effect.
- The Referee now suggests that we measure Circular Birefringence (CB). Unfortunately, CB is not well suited to study the current problem. CB arises from the difference in refractive index between right- and left-circularly polarized light, which applies when the eigenmodes of the system are purely circular. However, this definition is strictly valid only in the absence of linear birefringence. In the more general case, including the strained state considered here, both linear and circular birefringence are present simultaneously, and the eigenmodes become elliptically polarized. In this situation, the simple decomposition into purely right- and left-circular components no longer applies, and the optical response must instead be described in terms of coupled linear and circular contributions. The polarization-dependent measurements reported in our work directly probe this full response, providing access to both components, which are explicitly quantified. We

therefore emphasize that the circular birefringence referred to by the Referee is already inherently contained in our data.

Taken together, we conclude that the present results already provide compelling and conclusive evidence for the observation of intrinsic strain-induced chirality.

- A second concern is the claimed generality of the proposed mechanism. The authors argue that the piezochiral effect applies broadly to non-centrosymmetric materials. However, the specific mechanism demonstrated relies on the presence of compensated chiral substructures of opposite handedness within the unit cell, whose degeneracy is lifted by strain. It is not clear that such arrangements are common among non-centrosymmetric crystals. In many materials, chirality is either absent or already fixed globally, and there is no obvious internal degree of freedom that can be unbalanced in this way. While a coupling between strain and chirality may be symmetry-allowed in a formal sense, the physical realization illustrated here appears to be significantly more restricted than suggested.

The generality of the proposed mechanism is an important aspect of this work. We will show in the following discussion that the underlying scenario indeed applies to a broad class of systems.

In essence, the Referee's question reduces to whether local chiral substructures can, in general, be identified in systems where piezochiral coupling is symmetry-allowed. To address this point systematically, we consider the smallest structural unit of a crystal, the atomic site, which can already be chiral if its local environment lacks improper symmetry. Within this framework, a local chiral order parameter can be defined based on the electric toroidal monopole, as established in recent theoretical works (*Phys. Rev. Lett.* **129**, 116401 (2022); *J. Chem. Phys.* **160**, 184117 (2024).).

Following this argument, we analyze the local environments of each atomic site in the proposed systems based on the space group symmetries and the corresponding Wyckoff positions. As summarized in Table R2, all systems discussed in the manuscript host at least one atomic site with a locally chiral environment. Since these systems are globally achiral in the unstrained state, such local chirality must be compensated by symmetry-related counterparts of opposite handedness. Hence, these crystals can be viewed as being antiferrochiral and composed of local chiral substructures.

The application of strain breaks the symmetry operations that relate the opposite-handed local environments to each other. This lifts their degeneracy and renders them inequivalent, enabling the emergence of a net chiral response. Since all systems considered here exhibit locally chiral atomic sites, such compensated chiral substructures represent a general feature of this class of materials.

We thank the Referee for raising the point, which highlights the possibility of an alternative scenario where all atomic sites are statically achiral in a piezochiral system, an idea that is novel and of great interest. While this represents an intriguing direction for future work, we are currently not aware of material realizations of this type. We have added the corresponding table and analysis to the revised Supplementary Information to further elaborate on this point.

Crystal system	Point group	Space group	Chiral atomic sites
$AgGaS_2$	-42m	I-42d	S(8d)
γ-$LiBO_2$	-42m	I-42d	O(8d)
$KH2PO_4$	-42m	I-42d	H(8d), O(16e)
$ZnGeP_2$	-42m	I-42d	P(8d)
LiB_3O_5	mm2	$Pna2_1$	Li(4a), B(4a), O(4a)
$KTiPO_5$	mm2	$Pna2_1$	K(4a), Ti(4a), P(4a), O(4a)
Bi_2SeO_5	mm2	Aem2	O(4b), Bi(8d), Se(8d), O(8d)
SrB_4O_7	mm2	$Pmn2_1$	B(4b), O(4b)
BPO_4	-4	I-4	O(8g)
$CdAl_2S_4$	-4	I-4	S(8g)
NaI_3O_8	-4	P-4	I(2g), I(4h), O(4h)
$CsAs_4F_{13}$	-4	I-4	As(8g), F(8g)
$Cd(AlCl_4)_2$	m	Pc	Cd(2a), Al(2a), (2a)
$SnTa_2O_8$	m	Cc	Sn(4a), Ta(4a), O(4a)
$Te_2P_2O_9$	m	Cc	Te(4a), P(4a), O(4a)
$As_2O(SO_4)_2$	m	Pc	As(2a), O(2a), S(2a)

Table R2 | Chiral atomic sites in piezochiral crystal systems. All atomic sites with locally chiral environments are listed together with their corresponding Wyckoff positions. The same chemical element appearing at different Wyckoff positions denotes symmetry-inequivalent sites of that element within the crystal. The analysis is performed for the unstrained state.

- In summary, although the direction of the work is interesting, the experimental evidence for strain-induced optical activity is not yet sufficiently compelling, and the scope of the proposed effect is overstated. I therefore do not consider the manuscript suitable for publication in Nature in its present form.

We expect that our responses have successfully addressed the Referee's remaining concerns.

Response to Referee #2

- The authors have addressed all of my comments but one, i.e. a minor discrepancy regarding a promised acknowledgment that should be rectified before final acceptance.

We thank the Referee for the positive evaluation of our rebuttal. We will address the remaining point below.

- Below is a detailed response:

  - Focus on achiral systems: The authors provided a clear justification for focusing on achiral systems, noting that these materials allow for the deterministic switching between handedness by changing the direction or sign of applied strain.

  - As requested, the authors have included a detailed symmetry analysis in the revised Supplementary Information, explicitly listing the broken symmetry operations and the resulting chiral subgroups for various strain configurations and through a systematic classification of the piezochiral coupling for all point groups in the Supplementary Information, this is very good.

We thank the Referee for the earlier constructive suggestions, which have strengthened the paper and improved the clarity of the logical flow.

- - Literature integration: The authors included the relevant earlier works I mentioned (PRB 102, 235127 (2020) and J. Phys. Soc. Jpn. 43, 195-203 (1977)). However, in their rebuttal, the authors agreed to acknowledge that the term "piezochirality" was anticipated in a recent review (J. Phys.: Condens. Matter 37, 163004 (2025)). While they have added this paper as Reference 19, the current manuscript text uses the citation only to categorize the prior work as a limited existing approach that merely modulates"chirality, but this does not sounds to be associated to the anticipation I was mentionning. To fulfill their rebuttal promise, the authors should revise the wording at this citation to explicitly credit the conceptual anticipation of the term.

We understand the Referee's point. We have revised the corresponding passage in the manuscript to credit the prior anticipation of piezochirality in the literature. The revised text acknowledges the prior work more clearly and outlines the contribution of the present study.

- - In the limit of my understanding, the authors addressed the other referees' concerns by giving rigorous statistical validation through multi-wavelength and control experiments, performing a detailed symmetry analysis of the piezochiral pseudo-tensor, and clearly distinguishing their work from prior literature as a unique method for the deterministic selection of handedness.

The combination of rigorous symmetry analysis, first-principles simulations, and experimental validation in $AgGaS_2$ remains a very strong confirmation of the piezochiral effect.

We are pleased that the Referee finds our revisions satisfactory and recognizes that the concerns raised by the other Referees have been fully addressed, and that the combined experimental and theoretical results strongly support the piezochiral effect.

I recommend the manuscript for acceptance, providing the minor change with Reference 19 to accurately reflect the authors' rebuttal commitment regarding the anticipation of the "piezochirality" concept.

We sincerely appreciate the Referee's strong support for publication of our work in Nature.

Response to Referee #3

- After reading the comments of all the reviewers and the authors' convincing and instructive reply to them, I am now happy to recommend the manuscript for publication. There is one final point, the authors may like to take into account because it remains slightly confusing.

We thank the Referee for the supportive evaluation and for recommending the manuscript for publication in Nature. We address the remaining point as follows.

- When considering the chirality C = T(ij) epsilon(ij), then the use of group-theoretical tables in R. R. Birss, Symmetry and Magnetism, North-Holland 1966, reveals that C and T(ij) are axial i-tensors of even rank (0 and 2, respectively), whereas epsilon(ij) is a polar i-tensor of even rank (2). For the point group -42m we get for these (tables 7, 4b, 4d in Birss): C = 0, T(xx) = -T(yy), epsilon(xx) = epsilon(yy) and epsilon(zz). All other components are 0. In other words, the point group does not allow any chirality. The point is that the applied stress reduces the symmetry to a subgroup of -42m where the effect is allowed. This is exactly the point of this work. However, with this, statements like "in the point group -42m, the piezochiral coupling takes the form…" are misleading because once we have the strain, we are no longer in -42m. This confused me in my original report, and since the symmetry breaking is the key of the work, I suggest that the authors use more careful wording here.

We thank the Referee for raising this point. We have revised the manuscript to better distinguish between the symmetry of the unstrained state and the symmetry reduced by strain. The revised main text now emphasizes that chirality arises upon symmetry breaking induced by externally applied strain, which resolves the issue identified by the Referee.

Response to Referee #1

- The manuscript presents an interesting and potentially important idea. In particular, I find the central symmetry argument physically appealing. The proposed mechanism, in which strain lifts the compensation between opposite local chiral motifs and induces a net chiral response, appears conceptually general and worthy of broad attention. In my view, this mechanism is closely analogous to the emergence of a net ferromagnetic component when an external magnetic field perturbs a compensated antiferromagnetic state. This gives the work a clear conceptual significance beyond the specific material studied here.

We thank the Referee for the positive assessment of our work.

- At the same time, I remain concerned that the optical analysis does not yet reach the level of rigor that I would expect for publication in Nature.

  My main concern is not whether the observed signal is intrinsic in a broad phenomenological sense. The strain sign reversal, the orthogonal strain dependence, and the wavelength reproducibility together make a convincing case that the effect is not a trivial experimental artifact. Rather, my concern is more specific: whether the measured polarization rotation can be unambiguously interpreted as a direct measure of strain-induced optical activity itself.

Below, we provide additional analysis that directly addresses this point.

- The authors correctly point out in the rebuttal that, in the strained state, linear birefringence and gyrotropic effects coexist and the optical eigenmodes become elliptically polarized. However, once this is acknowledged, the interpretation of the measured rotation angle becomes substantially more subtle. In such a regime, the observed polarization evolution is generally not equivalent to a direct measurement of circular birefringence or optical activity. Instead, it reflects a coupled propagation problem involving birefringence, gyrotropic coupling, phase accumulation, sample thickness, and input polarization conditions.

  This issue has been analyzed carefully in the literature. In particular, Woodford et al. demonstrated that, in birefringent gyrotropic media, the observed polarization rotation can become a highly nontrivial function of propagation effects even in ideal optical systems where ordinary alignment artifacts are absent [S. R. Woodford, A. Bringer, and S. Blugel, J. Appl. Phys. 101, 053912 (2007)]. The important point is that these complications arise intrinsically from electromagnetic wave propagation itself, rather than from experimental imperfections.

By contrast, the present manuscript relies primarily on perturbative extraction procedures and angular averaging arguments. While these methods are reasonable as phenomenological analysis tools, they do not rigorously establish that the extracted quantity is uniquely proportional to the intrinsic gyrotropic tensor component. In particular, the rebuttal convincingly argues that ordinary birefringence artifacts are unlikely to dominate the signal, but this is not equivalent to demonstrating that the measured rotation directly equals intrinsic optical activity.

To address the Referee's specific concern, and to ascertain whether our experimental procedure provides a quantitatively reliable measure of optical activity in the presence of birefringence, we have performed numerical simulations that include both birefringence and optical activity at the same time.

Following the approach of Woodford et al. (J. Appl. Phys. 101, 053912 (2007)), we calculated the polarization rotation signal by decomposing the incident linearly polarized light into the elliptically polarized eigenmodes of the medium and recombining them at the sample output. The calculations were performed for our experimental conditions, using a probe wavelength of 808 nm, a sample thickness of 200 µm, and a static permittivity of 6.19, while scanning the incident probe polarization angle over 360°. The optical activity and birefringence are introduced by the parameters c and b in the permittivity tensor

$$\boldsymbol{\varepsilon} = \begin{pmatrix} \varepsilon_0 - b & ic \\ -ic & \varepsilon_0 + b \end{pmatrix}.$$

In the absence of birefringence ($b = 0$), the extracted rotation signal is a flat line independent of the incident polarization angle (Fig. R1a). As expected, the introduction of birefringence adds a polarization-angle-dependent modulation. Importantly, however, the polarization-averaged signal remains largely unaffected. The same holds for optical activity of the opposite sign (Fig. R1b). We quantified this effect further by calculating the polarization-averaged signal for a broad range of birefringence values (Fig. R1c). For the birefringence reached experimentally ($b \sim 5\times10^{-4}$), the deviation from the birefringence-free value is only ~1%.

These results provide clear evidence that, within the parameter range relevant to our measurements, birefringence introduces only a negligible correction to the extracted optical activity and the measured polarization rotation serves as an accurate quantitative measure of the strain-induced optical activity in the present study.

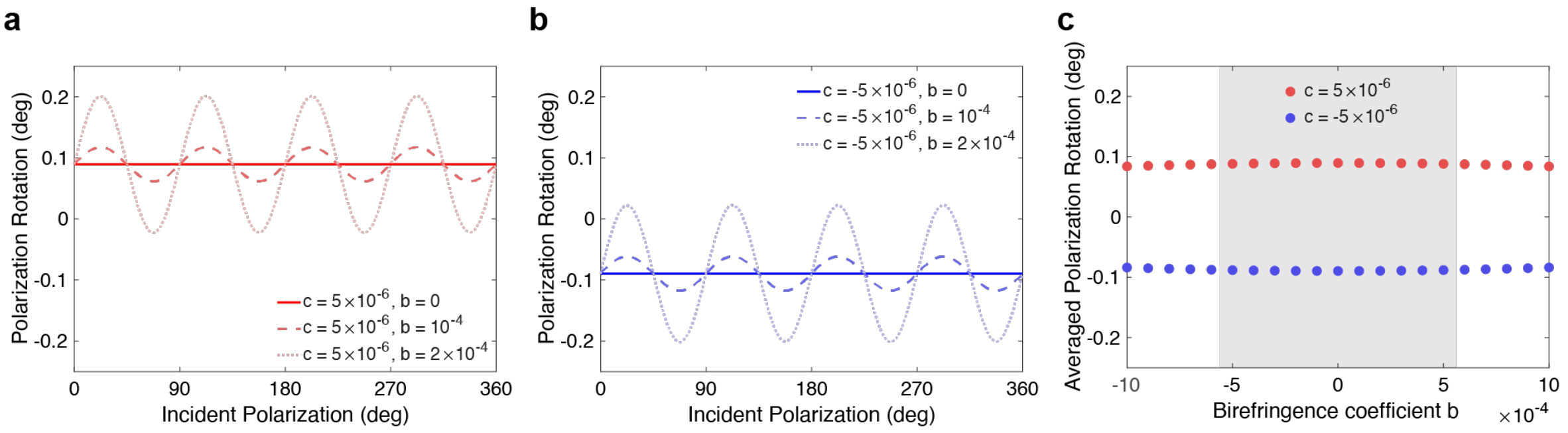

Fig. R1 | Simulated polarization rotation signal in the presence of both optical activity and birefringence. **a,** Polarization rotation signal as a function of incident polarization angle for positive optical activity, shown without and with birefringence. **b,** Same as in panel (a) but for optical activity of the opposite sign. **c,** Polarization-averaged rotation signal as a function of birefringence. The shaded region indicates the range of birefringence values reached in the experiment.

Lastly, we note that the strain-induced birefringence remains weak and enters only as a small perturbation, because the unstrained crystal is optically uniaxial along the probed direction and therefore doesn't show equilibrium birefringence. Indeed, the situation would be less favorable in materials with intrinsic birefringence, which is exactly the regime considered by Woodford et al..

- I also remain unconvinced by the way the manuscript positions ferroaxial approaches in comparison to the present mechanism. The authors repeatedly contrast their proposal with ferroaxial electric-field control and imply that the latter applies only to a relatively narrow class of materials. I do not think this comparison is sufficiently balanced. From a purely symmetry-based perspective, the difference in generality between the two approaches does not appear especially large: ferroaxiality is symmetry-allowed in 13 crystallographic point groups, whereas the linear piezochiral effect discussed here is allowed in 15 point groups. I therefore think the manuscript should moderate its comparative claims regarding the relative breadth or universality of the proposed approach.

We take the Referee's point. To avoid possible misunderstanding, we have revised the relevant discussion by removing a direct comparison to the earlier approaches.

- Overall, I consider the symmetry concept and the underlying physical picture to be interesting and potentially important. However, for a journal such as Nature, where the expected downstream influence of the claims is exceptionally high, I believe that statements concerning the direct observation and quantitative extraction of strain-induced optical activity should be supported by a more rigorous propagation analysis. At present, I

still see a gap between the demonstrated experimental phenomenology and the strength of the central optical interpretation.

We believe that our additional analysis and response above will allay the referee’s concerns.

Response to Referee #3

- When I consider the discussion between Reviewer 1 and the authors, it appears to me that the concerns raised by the Reviewer in the previous round have been addressed satisfactorily by the authors. What now remains are previously unraised doubts regarding the unambiguous experimental identification of chirality. A central issue is that the chiral response is intermixed with linear birefringence, and chirality is never measured independently of this background contribution. For the reviewer, the ambiguity of the experimental analysis is further reinforced by the fact that birefringence and chirality together give rise to a complex propagation behavior. I agree with the described phenomenology.

  The authors address this issue by conducting their analysis partly on a qualitative basis. The chirality manifests itself clearly through the investigated sign reversals and through their absence in non-chiral configurations. Certain effects of the birefringence average out in the analysis. For a qualitative demonstration of strain-induced chirality, I would regard this as sufficient, even though background-free chirality is not shown and the explicit quantitative propagation behavior resulting from the superposition of birefringence and chirality is not calculated. I would expect a fully quantitative treatment to appear in subsequent, more technically focused studies. This would also have to answer the question what chirality is worth when superimposed by a strong birefringent contribution.

  In addition to the analysis discussed above, the authors also demonstrate other consequences of the proposed strain dependence, such as a quadratic dependence and a characteristic dispersion behavior. Overall, I thus consider the reported results to provide convincing evidence for piezochirality.

We thank the Referee for these clarifying statements. They take the view that the experimental evidence presented in the manuscript provides convincing support for the existence of piezochirality.

In response to the concerns raised by Referee #1, we have provided additional numerical simulations that account for the combined effects of birefringence and optical activity during light propagation. These calculations clearly show that the effect of birefringence on the extracted optical activity is negligible within the experimentally relevant parameter range.

- The subsequent comments by Reviewer 1 concerning the comparison between ferroaxiality and piezochirality appear, according to the Reviewer's own wording, to be of secondary importance and mainly related to phrasing. A minor reformulation should be sufficient to resolve this issue.

In the revised manuscript, we have rephrased the relevant discussion to provide a clearer comparison between ferroaxiality and piezochirality.
